# Drug-induced agranulocytosis: a disproportionality analysis and umbrella review

**DOI:** 10.3389/fphar.2025.1641747

**Published:** 2025-09-17

**Authors:** Yajie Lu, Bin Wu, Kunyu Li, Zhonglin Liu, Yuxi Chen, Ting Xu

**Affiliations:** ^1^ Department of Pharmacy, West China Hospital, Sichuan University, Chengdu, Sichuan, China; ^2^ West China School of Pharmacy, Sichuan University, Chengdu, Sichuan, China

**Keywords:** drug-induced agranulocytosis, disproportionality analysis, umbrella review, FAERS, adverse drug event

## Abstract

**Background:**

Drug-induced agranulocytosis (DIA) is a rare but life-threatening hematologic disorder that demands increased clinical and research attention. This study aimed to provide the current overview of DIA for clinical guidance.

**Methods:**

Using real-world data from FDA Adverse Event Reporting System (FAERS), we performed a disproportionality analysis to identify the drugs associated with agranulocytosis, employing the information component and reporting odds ratio algorithms. Logistic analysis was conducted to explore the confounding factors of DIA. Time-to-onset analysis was implemented to compare the adverse event onset time among different drugs. To comprehensively supplement and corroborate our disproportionality findings, we further conducted an umbrella review of systematic reviews (SRs). Five electronic databases were searched with SRs addressing DIA as an included adverse event. Two independent reviewers performed literature screening, data extraction, and quality assessment according to the preferred reporting items for systematic reviews and meta-analysis statement. The results of the included SRs were synthesized using qualitative analysis.

**Results:**

The disproportionality analysis revealed that most identified DIA signals were for anticancer drugs. The top-five drugs with DIA signals by case number were methotrexate (6,462 cases), lenalidomide (5,722 cases), rituximab (5,691 cases), doxorubicin (4,391 cases), and carboplatin (4,371 cases). High-risk drugs (e.g., deferiprone), old age, and abnormal weight were strongly associated with DIA based on multivariate logistic regression. Time-to-onset analysis showed that clozapine has the longest median of onset time (1,121.3 days), while azithromycin has the shortest time (8.1 days). The umbrella review included seven systematic reviews, with five focusing on anticancer therapy. Their findings on DIA-associated drugs, including protein kinase inhibitors and immune checkpoint inhibitors, were consistent with those from the disproportionality analysis. Antibiotics, antithyroid drugs, and psychotropic drugs were also identified as causative drugs of DIA.

**Conclusion:**

This study systematically reviewed the FAERS database and existing literature on DIA to identify a spectrum of associated drugs. Anticancer drugs were predominant, with targeted therapies comprising a large proportion, while non-chemotherapy drugs were also identified as suspect drugs. These findings underscored the need for heightened clinical vigilance toward suspected drugs and highlighted the importance of future efforts to validate high-risk mechanisms and explore DIA monitoring strategies.

## Introduction

Agranulocytosis, the most severe form of neutropenia, is defined as an absolute neutrophil count (ANC) of <0.5 × 10^9^/L (Zhang et al., 2022). Internationally, its annual incidence ranges from 5.4 to 15.4 cases per million (Andrès et al., 2006). Since the concept of “agranulocytosis” was first proposed in 1922, researchers have gradually realized that agranulocytosis was mainly caused by drugs (Pisciotta, 1969), which is known as drug-induced agranulocytosis (DIA). Currently, it has been reported that patients with DIA typically present with fever and chills, sore throat, oral ulcers, and even fatigue, weakness, and severe infections (Rattay and Benndorf, 2021). Given that neutrophils are the primary effector cells of the innate immune system (Liew and Kubes, 2019), DIA confers a high risk of life-threatening complications. Studies showed that more than one-third of DIA patients developed septic shock or sepsis, and this proportion is doubled in the elderly populations (Andrès et al., 2008).

Patients usually exhibit no symptoms during the period of neutropenia. Drug discontinuation and symptomatic treatment after the onset of DIA symptoms often lead to poor prognosis (Andrès et al., 2008; Rattay and Benndorf, 2021). Therefore, vigilance and control of the occurrence and development of DIA is crucial in clinical practice. “Myeloid maturation arrest” and the presence of anti-neutrophil antibodies constituted key criteria for DIA diagnosis (Federici et al., 2008; Curtis, 2017). The main management measure for DIA involved monitoring the ANC levels in patients using suspected drugs (Tesfa et al., 2009). Therefore, identifying the suspicious drugs related to DIA is a prerequisite for implementing pharmacovigilance and management.

As a rare but severe adverse drug event (ADE), the landscape of DIA-associated drugs was mainly established through case reports and phase-IV clinical trials, which limited the breadth of drug identification (Pisciotta, 1978; Andersohn F. et al., 2007; Garbe, 2007; Aguilar-Guisado et al., 2017). To address this situation, real-world evidence (RWE) served as a valuable supplement, with a wide range of data sources that enabled it to reflect potential ADE in different populations and various clinical settings (Azoulay, 2022). However, the RWE study on DIA was still limited in the past decade (Wu et al., 2025). The FDA Adverse Event Reporting System (FAERS), a real-world data set, played an important role in pharmacovigilance for the large repository of ADEs and frequent updates (Yu et al., 2021). Moreover, umbrella review—a methodology rooted in evidence-based medicine (EBM)—emerged as a critical complement to RWE by synthesizing evidence from multiple systematic reviews (SRs) (Dreyer, 2022). It could systematically appraise whether RWE signals align with consistent findings across clinical trials, observational studies, and case reports documented in published SRs.

In the current study, we performed a comprehensive disproportionality analysis to identify the drugs associated with agranulocytosis events. Additionally, we conducted an umbrella review to integrate safety data from existing SRs and enhance the robustness and generalizability of the pharmacovigilance results. The aim of the present study was to present a complete picture of drugs that could induce agranulocytosis and provide reference data for further study and clinical practice.

## Methods

### The disproportionality analysis

The disproportionality analysis was conducted following the Reporting of a Disproportionality Analysis for Drug Safety Signal Detection Using Individual Case Safety Reports in PharmacoVigilance (READUS-PV) guidelines (Supplementary Material S1, S2) (Fusaroli et al., 2024).

#### Data source and study design

The retrospective pharmacovigilance study collected data from the FAERS Quarterly Data Extraction website between the first quarter of 2004 and the fourth quarter of 2023. The FAERS datasets contained demographic information, drug information, and reaction information. Each report contained at least one suspected drug and one or more ADE terms. The downloaded data were imported into SQL server software, and we established our own convenient and directly usable database. Data cleaning procedures were as follows: when the CASEID (the number used to identify FAERS cases) and FDA_DT (the date when the FDA received the case) were the same, the data unit with the latest FDA_DT was kept in the DEMO table for de-duplication, and then the “deleted” cases were removed. The entire process of de-duplication was based on recommendations by the FDA.

#### Data identification

The ADE terms in the FAERS database were based on the International Medical Terminology Dictionary for Regulatory Activities (MedDRA). The DIA-related preferred terms (PTs) in the MedDRA structure were investigated according to the standardized MedDRA query (SMQ) narrow search “agranulocytosis.” The resultant 15 PTs and PT_code are listed in Supplementary Table S1.

The generic names, brand names, synonymous names, or abbreviations contribute to the various drug names in the FAERS database. We used MedEx software (MedEx UIMA 1.3.8, Vanderbilt University, US) to convert different forms of drug names into the “generic name” for standardization. The potential target drugs were counted on the basis of the “generic name,” and the primary suspected (PS) drugs were included for further analysis.

#### Disproportionality analysis

As agranulocytosis cases were identified, drugs with case number >10 were included for further analysis. Frequency difference between specific drug event combination (DEC) and the background was investigated following the disproportionality principle. It was considered a significant signal when the DEC frequency was higher than the background frequency and within the set threshold, which revealed a statistical correlation between the target drug and the target ADE.

The reporting odds ratio (ROR), a frequency-based method, was one of the most commonly used disproportionality algorithms. A greater ROR value indicates a stronger signal, signifying a robust statistical relationship between the target drug and ADE. An ROR signal was identified when at least three cases were reported with the lower limit of 95% confidence interval (CI) of ROR >1. In addition to the ROR approach, this study incorporated the information component (IC) method, a Bayesian method, to detect signals of disproportionality. An IC signal was detected when the lower limit of 95% CI > 0. Both the methods were regularly utilized for the identification of disproportionality signals, with the IC method demonstrating superior sensitivity and specificity, particularly in datasets with smaller sample sizes. In this study, the DIA signal was identified through the generation of both the ROR and IC signals.

#### Logistic regression

FAERS reports containing patient information (gender, age, and weight) were extracted, and only those with complete data were analyzed. Age >120 years and weight >400 kg were defined as outliers and excluded from the analysis.

A univariable logistic analysis for suspicious drugs was conducted using the following parameters: 95% CI lower limit of ROR > l, case number >100, and p-value <0.01. As a result, drugs with p <0.01 were further used for least absolute shrinkage and selection operator (LASSO) regression (Zhang et al., 2023). Multivariate logistic regression was able to determine the existence of DIA risk factors using the drugs screened by cross-validation LASSO (CV-LASSO) combined with basic patient information as independent variables. After constructing a multivariate logistic regression model, we used the receiver operating characteristic (ROC) curve to calculate the area under the curve (AUC) value for assessing the accuracy of the model’s predictions. When 0.5< AUC <1, the larger AUC value was regarded as a better classifier of the model.

#### Time-to-onset time analysis

The medications would be systematically classified for further analysis according to the multivariate logistic regression results.

The onset time was defined as the interval between EVENT_DT (date of adverse event occurrence) and START_DT (start date for drug use). Moreover, the input errors (EVENT_DT earlier than START_DT) reports or inaccurate date entries were excluded. Therefore, the drug-induced time was the difference between the two aforementioned time points and depicted as the cumulative curve and violin plot.

R software (version 4.2.1) was used for logistic regression and time-to-onset analysis.

### The umbrella review

The umbrella review was conducted in accordance with the Preferred Reporting Items for Systematic Reviews and Meta-Analyses (PRISMA) guidelines (Supplementary Material S3) (Page et al., 2021). The protocol was registered with PROSPERO (CRD420251037098).

#### Search strategy and eligibility criteria

We systematically searched PubMed, Embase, The Cochrane Library, and Web of Science from the inception to April 2025. For pharmacovigilance, case reports serve as a critical source for identifying rare and serious ADE. Therefore, SRs of randomized clinical trials (RCTs), observational studies, and case reports were included. The whole search strategy was provided in Supplementary Table S2. In brief, we utilized the controlled term “agranulocytosis” combined with free terms such as “neutropenia.” No language limits were applied in the search strategy.

Regarding the Population, Intervention, Comparison, Outcome, and Study design (PICOS) framework, there were no limitations in population, intervention, and comparison. Consistent with the FAERS study, we included SRs that clearly investigated the association between any intervention and agranulocytosis. The excluded criteria were as follows: not full-text report, grade-1/2 neutropenia, grade not reported, congenital neutropenia, neutropenia developed before therapy, and reported outcome not identified as severe neutropenia or agranulocytosis.

#### Study screening, data extraction, and quality assessment

Two authors (YL and KL) screened each record independently with discrepancies, and conflicts were settled by the third author (BW). Relevant data such as the author, year, time range, number of studies included, number of patients with DIA, interventions, and outcomes were extracted and listed in a Microsoft Excel document by YL and meticulously reviewed by KL.

The methodological quality of the included SRs was assessed in accordance with the Assessment of Multiple Systematic Reviews 2 (AMSTAR-2) (Shea et al., 2017). Following the guidance document of AMSTAR-2, the 16 items in the questionnaire were completed by YL and KL independently, and then the overall quality of each study was generated based on critical weaknesses. Meanwhile, any disagreement regarding the AMSTAR-2 scale was resolved by BW to reach consensus.

#### Data synthesis

To obtain safety information comprehensively, SRs of all research types were included, so quantitative synthesis was not applicable in this study. As part of qualitative evaluation, the synthesis was structured thematically around drug classes, aligning with the classification framework used in the disproportionality analysis to facilitate cross-validation. We systematically extracted and synthesized key findings from the included SRs, including the cumulative incidence, effect size of relative risk (RR) with their CI, heterogeneity, and discrepancies.

## Results

### The disproportionality analysis

#### The primary characteristics of DIA cases

**FIGURE 1:**
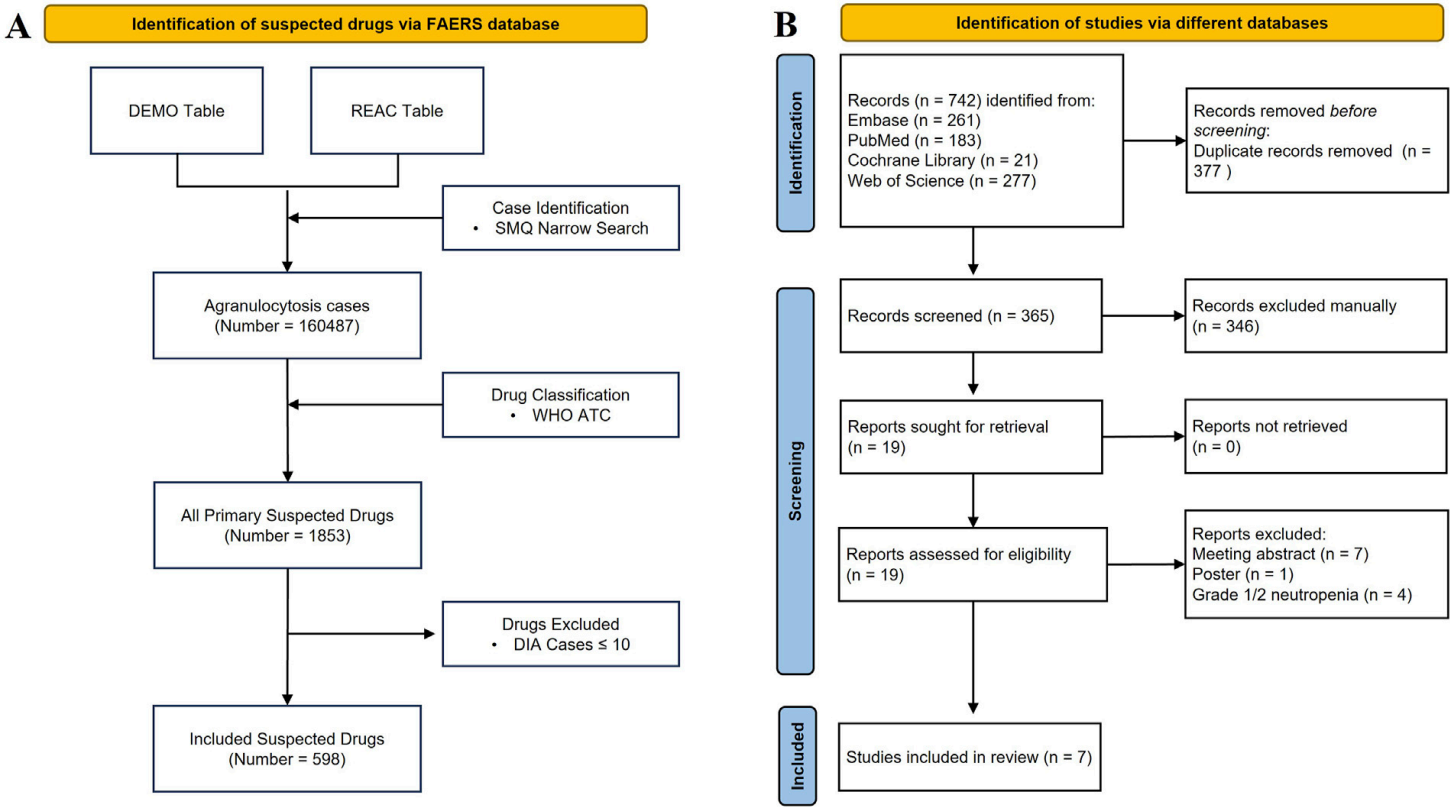
Flowchart for the pharmacovigilance study and umbrella review. **(A)** Identification of the DIA cases from FAERS. **(B)** PRISMA diagram for the selection of systematic reviews.

**FIGURE 2:**
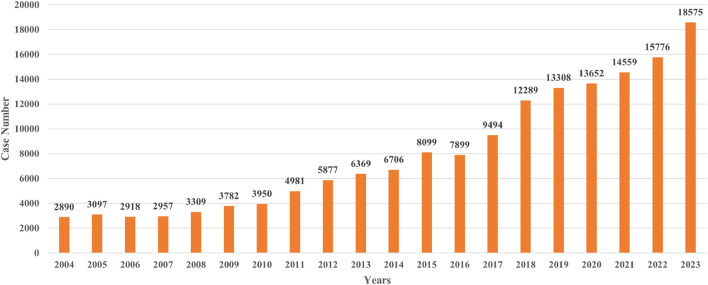
Annual reported DIA cases in FAERS from 2004 to 2023.

A total of 160,487 agranulocytosis cases with PS drugs were identified, accounting for 0.92% of all ADE cases in FAERS. The PS drugs of each report were consolidated, resulting in 1,853 PS drugs. Then, 598 PS drugs with more than 10 cases were included and analyzed (Figure 1A). The annual numbers of reports from 2004 to 2023 are shown in Figure 2.

The average age of the reported cases was 54.79 ± 21.69 years, and the other characteristics of the patients, including basic information such as gender, outcome, the reporter’s profession, and reporter’s region, are shown in Supplementary Table S3. The number of female patients (71,422, 44.50%) was higher than that of male patients (68,183, 42.49%). A total of 137,500 cases (85.68%) were reported by health professionals. The top region of reporting was Europe (58,051, 36.17%). There were 19,679 (12.26%) death cases and 10,384 (6.47%) life-threatening cases.

#### DIA signal detection

A total of 251 drugs with more than ten cases were identified with DIA signals. We classified the positive DIA signal drugs according to the WHO Anatomical Therapeutic Chemical (ATC) system (Supplementary Table S4). The top-five drug categories warranting attention were monoclonal antibodies and antibody–drug conjugates (ATC code: L01F, 23,656 cases, 35 drugs), immunosuppressants (ATC code: L04A, 21,099 cases, 21 drugs), other antineoplastic agents (ATC code: L01X, 17,345 cases, 33 drugs), antimetabolites (ATC code: L01B, 14,869 cases, 13 drugs), and plant alkaloids and other natural products (ATC code: L01C, 9,230 cases, 11 drugs), as shown in Supplementary Table S5.

According to the WHO ATC classification, we reported the top-50 drugs by the case number (Table 1) and the top-50 drugs by the signal strength (Table 2) in the main text. The top-five drugs by case number were methotrexate (6,462 cases), lenalidomide (5,722 cases), rituximab (5,691 cases), doxorubicin (4,391 cases), and carboplatin (4371 cases). The top-five drugs by signal strength were toripalimab (ROR = 73.93; 95% CI 45.26–120.77), mesna (ROR = 46.16; 95% CI 38.52–55.32), cytarabine (ROR = 43.32; 95% CI 41.55–45.17), dexrazoxane (ROR = 42.55; 95% CI 33.50–54.05), and clofarabine (ROR = 38.63; 95% CI 35.23–42.36).

**TABLE 1:** Signal detection of drug-induced agranulocytosis (top-50 drugs by case number).

Category	Drug	DIA case number (N)	ROR	95% CI for ROR	IC	95% CI for IC	Daily med	EMC	Frequency/EMC
Immunosuppressants	Methotrexate	6,462	6.35	6.19–6.52	2.55	2.46–2.63	√	√	Uncommon
	Lenalidomide	5,722	1.97	1.92–2.02	0.94	0.85–1.03	√	√	Very common
	Mycophenolic acid	1,619	3.35	3.19–3.52	1.70	1.54–1.87	√	√	Not known
	Cyclosporine	1,145	2.83	2.67–3	1.47	1.27–1.66			
	Tacrolimus	983	1.97	1.85–2.1	0.96	0.75–1.17	√	√	Not known
	Pomalidomide	978	1.48	1.39–1.58	0.56	0.35–0.77	√	√	Very common
	Eculizumab	942	2.17	2.03–2.31	1.10	0.88–1.31			
Monoclonal antibodies and antibody–drug conjugates	Rituximab	5,691	7.47	7.27–7.67	2.77	2.68–2.86	√		
	Bevacizumab	2,896	5.19	5–5.39	2.30	2.17–2.42	√		
	Trastuzumab	2,199	8.90	8.52–9.3	3.04	2.89–3.18	√		
	Pembrolizumab	1,239	3.45	3.26–3.65	1.74	1.55–1.93	√	√	Very common
	Atezolizumab	971	5.81	5.45–6.2	2.47	2.25–2.68	√		
	Nivolumab	863	1.55	1.45–1.65	0.62	0.39–0.84	√	√	Uncommon
	Cetuximab	807	4.56	4.25–4.89	2.14	1.9–2.36	√		
	Obinutuzumab	798	15.59	14.47–16.8	3.77	3.51–4	√	√	Very common
	Pertuzumab	781	14.54	13.49–15.67	3.69	3.42–3.91	√	√	Very common
Antimetabolites	Capecitabine	1,749	3.58	3.41–3.76	1.80	1.63–1.95	√		
	Fluorouracil	1,506	9.65	9.15–10.17	3.15	2.96–3.32	√		
	Pemetrexed	1,299	14.42	13.6–15.28	3.67	3.47–3.85	√	√	Not known
	Cytarabine	3,135	43.32	41.55–45.17	4.94	4.79–5.06	√		
	Azacitidine	2,439	20.93	20.04–21.86	4.13	3.97–4.26	√	√	Very common
	Gemcitabine	1,800	8.52	8.12–8.95	2.98	2.82–3.14	√	√	Common
	Fludarabine	1,122	18.75	17.59–19.98	4.00	3.77–4.19	√	√	Very common
Other antineoplastic agents	Carboplatin	4,371	14.14	13.7–14.6	3.62	3.51–3.72	√		
	Venetoclax	2,593	8.80	8.45–9.16	3.02	2.88–3.15		√	Very common
	Cisplatin	2,213	15.53	14.85–16.25	3.76	3.6–3.9	√		
	Oxaliplatin	1,266	4.98	4.71–5.27	2.26	2.06–2.44		√	Very common
	Bortezomib	1,223	4.63	4.37–4.9	2.15	1.96–2.34		√	
Plant alkaloids and other natural products	Docetaxel	2,132	4.86	4.66–5.08	2.22	2.07–2.36	√	√	Very common
	Etoposide	1,898	23.10	21.97–24.28	4.25	4.07–4.4	√		
	Paclitaxel	1,790	6.17	5.88–6.48	2.55	2.38–2.7	√	√	Common
	Vincristine	1,595	31.48	29.77–33.3	4.61	4.4–4.77	√		
	Irinotecan	792	7.48	6.96–8.04	2.81	2.56–3.04	√	√	Very common
Alkylating agents	Cyclophosphamide	3,965	18.39	17.77–19.03	3.96	3.84–4.06	√	√	Not known
	Temozolomide	1,311	9.73	9.19–10.3	3.16	2.96–3.34	√	√	Very common
	Bendamustine	878	11.76	10.97–12.61	3.41	3.17–3.63	√		
	Melphalan	820	16.75	15.56–18.04	3.86	3.6–4.08	√		
Cytotoxic antibiotics and related substances	Doxorubicin	4,391	18.51	17.92–19.12	3.96	3.85–4.06	√	√	Common
	Epirubicin	770	15.82	14.66–17.06	3.79	3.52–4.02	√	√	
Protein kinase inhibitors	Palbociclib	2,173	3.36	3.21–3.5	1.70	1.56–1.84	√	√	Very common
	Imatinib	1,308	2.69	2.54–2.84	1.40	1.21–1.58	√	√	Not known
	Ruxolitinib	861	1.71	1.6–1.83	0.76	0.54–0.98	√	√	Common
	Sunitinib	756	2.27	2.11–2.44	1.16	0.92–1.4	√	√	Not known
Corticosteroids	Dexamethasone	2,208	6.77	6.49–7.07	2.67	2.52–2.81			
	Prednisolone	972	3.53	3.31–3.77	1.78	1.56–1.99			
	Prednisone	851	3.16	2.95–3.38	1.63	1.4–1.85			
Immunostimulants	Pegfilgrastim	2,363	3.46	3.32–3.6	1.74	1.6–1.88			
	Filgrastim	1,040	11.22	10.52–11.96	3.35	3.12–3.55			
Antipsychotics	Clozapine	2069	2.52	2.41–2.63	1.30	1.15–1.45	√	√	Uncommon
Other antibacterials	Linezolid	944	9.02	8.44–9.64	3.06	2.83–3.27	√		

DIA, drug-induced agranulocytosis; CI, confidence interval; N, number of reports, ROR, reporting odds ratio; IC, information component; WHO ATC, WHO anatomical therapeutic chemical (ATC) system.

√ in DailyMed means that there are reports or cases showing a relationship between the drug and agranulocytosis. √ in EMC means that the target drug has agranulocytosis or grade-4 neutropenia as an adverse reaction in its drug label. Frequency/EMC means the target drug frequency in its EMC drug label is very common (≥1/10), common (≥1/100 to <1/10), uncommon (≥1/1,000 to <1/100), rare (≥1/10,000 to <1/1,000), or very rare (<1/10,000).

**TABLE 2:** Signal detection of drug-induced agranulocytosis (top-50 drugs by signal strength [ROR value]).

Category	Drug	DIA case number (N)	ROR	95% CI for ROR	IC	95% CI for IC	Daily Med	EMC	Frequency/EMC
Monoclonal antibodies and antibody–drug conjugates	Toripalimab	27	73.93	45.26–120.77	5.46	2.64–5.58	√		
	Gemtuzumab ozogamicin	496	34.25	30.95–37.90	4.71	4.31–4.96	√	√	Very common
	Polatuzumab vedotin	212	16.09	13.92–18.59	3.82	3.26–4.20	√	√	Not known
	Obinutuzumab	798	15.59	14.47–16.80	3.77	3.51–4.00	√	√	Very common
	Pertuzumab	781	14.54	13.49–15.67	3.69	3.42–3.91	√	√	Very common
Detoxifying agents for antineoplastic treatment	Mesna	168	46.16	38.52–55.32	5.02	4.20–5.35			
	Dexrazoxane	94	42.55	33.50–54.05	4.94	3.79–5.30	√		
	Trilaciclib	21	16.37	10.34–25.91	3.84	1.67–4.63	√		
Antimetabolites	Cytarabine	3,135	43.32	41.55–45.17	4.94	4.79–5.06	√	√	Common
	Clofarabine	616	38.63	35.23–42.36	4.84	4.48–5.07	√	√	Common
	Tioguanine	112	36.93	29.79–45.79	4.79	3.80–5.17			
	Decitabine	598	24.25	22.18–26.50	4.31	3.98–4.56	√	√	Very common
	Azacitidine	2,439	20.93	20.04–21.86	4.13	3.97–4.26	√	√	Very common
	Mercaptopurine	366	19.06	17.05–21.30	4.03	3.60–4.33	√	√	Very common
	Fludarabine	1,122	18.75	17.59–19.98	4	3.77–4.19	√	√	Very common
	Nelarabine	66	16.17	12.48–20.95	3.82	2.72–4.41	√	√	Very common
Cytotoxic antibiotics and related substances	Idarubicin	292	38.61	33.77–44.14	4.84	4.28–5.13		√	Very common
	Daunorubicin	221	28.03	24.17–32.51	4.48	3.87–4.83		√	Not known
	Mitoxantrone	334	24.1	21.40–27.14	4.31	3.84–4.62	√	√	Very common
	Dactinomycin	132	21.05	17.47–25.37	4.15	3.37–4.59	√		
	Doxorubicin	4,391	18.51	17.92–19.12	3.96	3.85–4.06	√	√	Common
	Bleomycin	209	18.14	15.66–21.00	3.97	3.39–4.35			
	Epirubicin	770	15.82	14.66–17.06	3.79	3.52–4.02	√		
Protein kinase inhibitors	Gilteritinib	586	38.06	34.63–41.82	4.82	4.45–5.06	√	√	Not known
NA	Lestaurtinib	24	37.69	23.67–60.03	4.81	2.28–5.21			
Alkylating agents	Pipobroman	14	37.37	20.33–68.69	4.8	1.42–5.21			
	Ifosfamide	529	23.82	21.67–26.17	4.29	3.93–4.55	√	√	Not known
	Thiotepa	225	22.07	19.12–25.48	4.2	3.63–4.57			
	Cyclophosphamide	3,965	18.39	17.77–19.03	3.96	3.84–4.06	√	√	Not known
	Melphalan	820	16.75	15.56–18.04	3.86	3.60–4.08			
Other analgesics and antipyretics	Metamizole sodium	22	36.71	22.61–59.59	4.79	2.14–5.20			
Other antineoplastic agents	Cytarabine and daunorubicin	262	34.72	30.20–39.92	4.72	4.14–5.04	√		
	Eribulin	699	26.32	24.22–28.59	4.41	4.10–4.64	√	√	Very common
	Vorinostat	211	19.23	16.61–22.27	4.04	3.46–4.42			
	Pegaspargase	579	18.97	17.36–20.73	4.02	3.69–4.27	√	√	Very common
	Cisplatin	2,213	15.53	14.85–16.25	3.76	3.60–3.90	√		
Immunostimulants	Colony stimulating factors	25	34.23	21.81–53.71	4.71	2.30–5.15			
Plant alkaloids and other natural products	Vincristine	1,595	31.48	29.77–33.30	4.61	4.40–4.77	√		
	Trabectedin	220	23.62	20.41–27.33	4.29	3.69–4.64	√	√	Very common
	Etoposide	1,898	23.1	21.97–24.28	4.25	4.07–4.40	√		
	Topotecan	482	17.96	16.30–19.78	3.95	3.59–4.23	√		
	Vinorelbine	290	15.64	13.83–17.69	3.78	3.31–4.12	√	√	Very common
Antigout preparations	Colchicine and probenecid	20	25.43	15.61–41.41	4.37	1.85–4.97		√	Not known
Therapeutic radiopharmaceuticals	Ibritumomab tiuxetan (90Y)	160	22.77	19.20–27.01	4.24	3.53–4.65	√		
	Strontium (89Sr) chloride	34	17.63	12.26–25.34	3.93	2.26–4.61			
	Samarium sm-153	12	15.08	8.24–27.59	3.74	0.85–4.69			
Antithyroid preparations	Thiamazole	241	18.12	15.81–20.77	3.97	3.43–4.33			
Antiinfectives	Chloramphenicol	21	16.25	10.27–25.72	3.83	1.67–4.63		√	Not known
Direct acting antivirals	Ganciclovir	225	16.04	13.94–18.45	3.81	3.27–4.19	√	√	Rare
Immunosuppressants	Antithymocyte immunoglobulin (rabbit)	13	15.09	8.44–26.97	3.74	0.97–4.67			

DIA, drug-induced agranulocytosis; CI, confidence interval; N, number of reports, ROR, reporting odds ratio; IC, information component; WHO ATC, WHO anatomical therapeutic chemical (ATC) system.

√ in DailyMed means that there are reports or cases showing a relationship between the drug and agranulocytosis. √ in EMC means that the target drug has agranulocytosis or grade-4 neutropenia as an adverse reaction in its drug label. Frequency/EMC means the target drug frequency in its EMC drug label is very common (≥1/10), common (≥1/100 to <1/10), uncommon (≥1/1,000 to <1/100), rare (≥1/10,000 to <1/1,000), or very rare (<1/10,000).

Notably, since we defined a positive signal when both the ROR and IC signals were generated, we excluded 52 drugs. These drugs had positive ROR signals but negative IC signals. For example, tislelizumab (ROR = 4.45, 95% CI 2.43–8.13; IC = 2.11, 95% CI -0.17–3.67), diazoxide (ROR = 4.26, 95% CI 2.39–7.58; IC = 2.05, 95% CI -0.11–3.57), norfloxacin (ROR = 3.58, 95% CI 1.96–6.53; IC = 1.81, 95% CI -0.38–3.44), and other drugs could not confirm the association with agranulocytosis.

#### Risk factors for DIA

**FIGURE 3:**
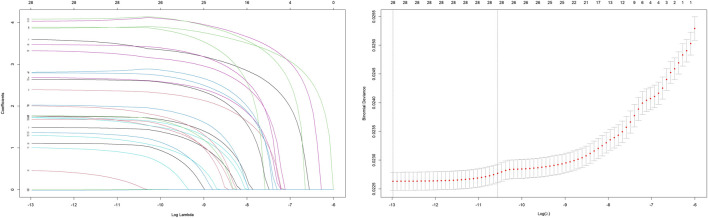
Selecting the risk factors of DIA by LASSO regression.

**FIGURE 4:**
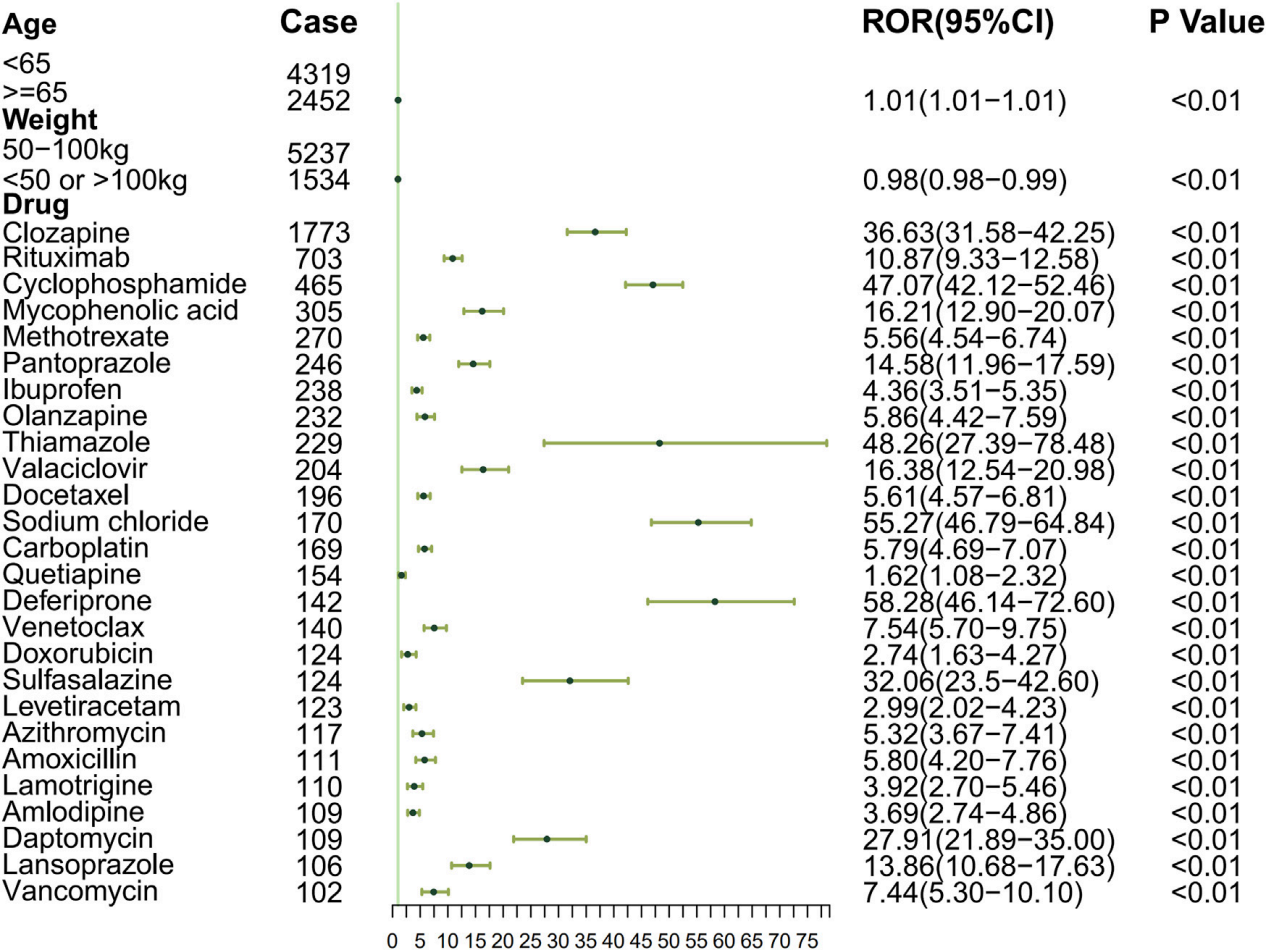
Forest plot of the logistic regression model in DIA.

**FIGURE 5:**
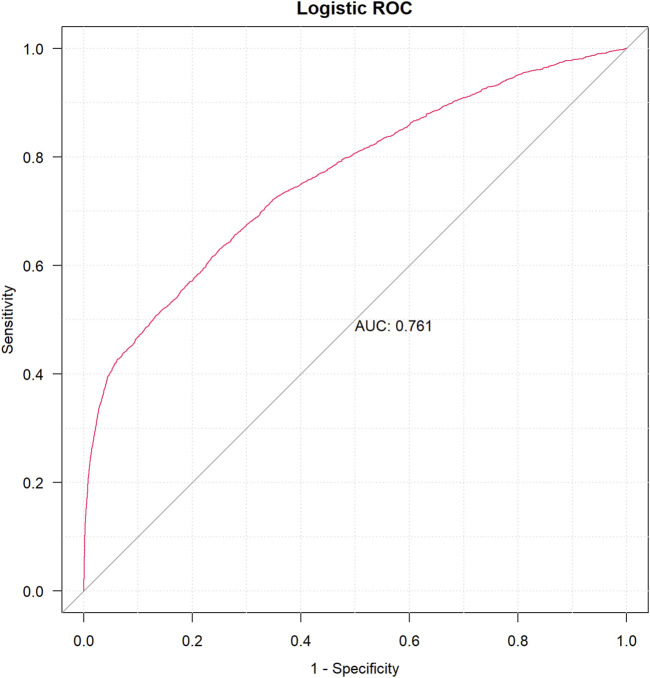
ROC curve of the logistic regression model.

To systematically evaluate the drug–event associations, we initially performed univariable logistic regression on 27 pharmacologically active compounds identified through univariate screening. Subsequently, LASSO regression with 10-fold CV-LASSO was applied to these candidate drugs and potential confounding factors (age, weight, and gender). The optimal penalty parameter λ selected 26 significantly associated drugs, along with age and weight as meaningful covariates (Figure 3). As shown in Figure 4, a multivariable logistic regression model incorporating these selected variables demonstrated statistically significant associations for all included parameters (p < 0.01). Deferiprone, thiamazole, cyclophosphamide, and clozapine are strongly associated with DIA. The accuracy of the model was validated by ROC curve analysis, yielding an AUC of 0.761, indicating satisfactory predictive accuracy (Figure 5).

#### Time-to-onset time analysis for DIA

Based on the multivariate logistic regression results, the medications were systematically classified into seven subgroups for comparative analysis of the temporal patterns in adverse reaction occurrence.

**FIGURE 6:**
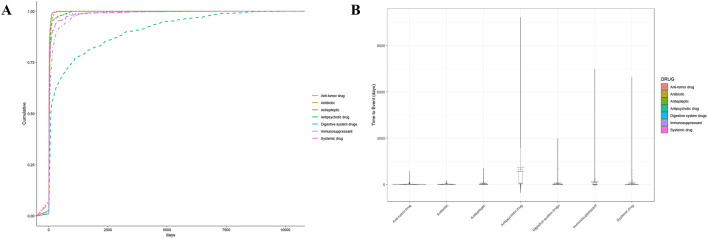
Time-to-onset time analysis for DIA. **(A)** Cumulative curve of DIA by different systems. **(B)** Violin plot of DIA by different systems. Subgroups: antipsychotic drug (clozapine, olanzapine, and quetiapine), immunosuppressants (rituximab, cyclophosphamide, mycophenolic acid, methotrexate, and sulfasalazine), antibiotics (azithromycin, amoxicillin, vancomycin, daptomycin, and valaciclovir), anti-tumor drugs (docetaxel, carboplatin, doxorubicin, and venetoclax), antiepileptics (levetiracetam and lamotrigine), digestive system drugs (pantoprazole and lansoprazole), and systemic drugs (thiamazole, deferiprone, amlodipine, sodium chloride, and ibuprofen).

Then, we performed a statistical analysis of the association between the duration of drug use and DIA. The results in Supplementary Table S6 and Figure 6 reveal significant differences in the duration of use. Clozapine, an antipsychotic drug, had the longest onset time (mean: 1,121.3, Q1: 51.0, Q3: 1,331.0). In contrast, antibiotic drugs such as amoxicillin and azithromycin had relatively shorter usage durations, averaging 11.0 days and 8.1 days, respectively, with lower associations with agranulocytosis. Chemotherapeutic agents, such as carboplatin, had an average duration of 18.0 days, with a certain level of association with agranulocytosis.

### The umbrella review

#### Literature search and characteristics of the included studies

A total of 742 records were identified, which included 261 from Embase, 183 from PubMed, 21 from Cochrane Library, and 277 from Web of Science. After 377 duplicates were removed, 365 records were left to be screened. Of these, we excluded 346 records at title/abstract screening, eight at full-text, and four at grade 1/2 or not reported, resulting in seven studies (Andersohn F. et al., 2007; Lyman et al., 2016; Kassem et al., 2018; Omar et al., 2020; Holz J.M. et al., 2022; Jiang X. et al., 2022; Moinard-Butot F. et al., 2023) being included for further review (Figure 1B). The records excluded along with the specific reasons are listed in Supplementary Table S7.

Table 3 summarizes the key characteristics of included SRs examining DIA events, which spanned a broad time range from 1966 to 2023. The included SRs investigated a range of interventions, encompassing tyrosine kinase inhibitors, CDK4/6 inhibitors, immune checkpoint inhibitors (ICIs), chemotherapeutic regimens, antibiotics, and other agents such as antithyroid drugs and psychotropics. The majority of the outcomes described in these studies was agranulocytosis, with or without severe complications. Among these SRs, three included only RCTs, another three included solely case reports, and just one incorporated both RCTs and observational studies. Sample sizes varied greatly from 83 to 8,593 patients, which is largely attributed to the difference in the types of included research.

**TABLE 3:** Characteristics of the included systematic reviews.

Author, year	Time range	Number of included studies	Intervention	Number of patients with DIA	Outcome	Study quality
Fabien, 2023	−2023	27	Crizotinib	501	Grade 3/4 neutropenia	Critically low
		11	Alectinib	26		
		7	Ceritinib	14		
		1	Brigatinib	3		
		2	Lorlatinib	1		
		3	Entrectinib	12		
Jiang, 2022	−2022	11	Sunitinib	173	Grade 3/4 neutropenia	Moderate
		1	BSC + Sunitinib	2		
Loay, 2018	2010–2017	2	Palbociclib + Letrozole	338	Grade 3/4 neutropenia	Low
		1	Fulvestrant + Palbociclib	214		
		1	Ribociclib + Letrozole	198		
		1	Fulvestrant + Abemaciclib	117		
		1	Abemaciclib + NSAI	69		
Gary H, 2016	2005–2015	36	Standard CHOP - like	33	Grade 3/4 neutropenia	Low
		11	Intense CHOP - like	10		
		5	CHOEP - like	5		
		5	ACVBP - like*	5		
		5	CVP	5		
		3	MCP	3		
		14	Single agent	13		
		13	Other regimens	11		
Nabil, 2020	2008–2020	21	Nivolumab	3	Severe neutropenia with or without complications	Low
		15	Ipilimumab	2		
Julian, 2022	1968–2020	29	Penicillin	28	Severe (<500), with or without neutropenic sepsis	Critically low
		19	Non-penicillin beta-lactams	19		
		28	Glycopeptides	19		
Frank, 2007	1966–2006	672	Analgesics and nonsteroidal anti-inflammatory drugs	29	Agranulocytosis with sepsis, non-systemic infection, or no infection	Low
			Antiarrhythmics	39		
			Anti-infective drugs	113		
			Anticonvulsants	10		
			Antineoplastics	33		
			Antirheumatics	28		
			Antithyroid drugs	87		
			Cardiovascular drugs	35		
			Gastrointestinal drugs	19		
			Psychotropic drugs	85		
			Other drugs	16		

Note. BSC: best supportive care: NSAI: non-steroidal aromatase inhibitor; CHOP: cyclophosphamide, doxorubicin, vincristine, and prednisone; CHOEP: etoposide, cyclophosphamide, doxorubicin, vincristine, and prednisone; ACVBP: doxorubicin, cyclophosphamide, vindesine, bleomycin, and prednisone; CVP: cyclophosphamide, vincristine, and prednisone; MCP: mitoxantrone, chlorambucil, and prednisolone; * includes 50% more doxorubicin and cyclophosphamide than CHOP/CHOEP.

#### Quality of included studies

The quality of the included SRs assessed by the AMSTAR-2 scale was moderate in one, low in four, and critically low in two. Four SRs failed to satisfy the critical domains that pertain to the consideration of risk-of-bias (RoB) in individual studies during the interpretation and discussion of review results (Supplementary Table S8).

#### DIA of anti-cancer therapy drugs

Five studies focused on cancer treatment, three of which investigated protein kinase inhibitors (PCIs). As the first-generation anaplastic lymphoma kinase (ALK) inhibitor, crizotinib-induced grade 3/4 neutropenia was reported in 0%–30% of patients with lung cancer, while the rate of the second-/third-generation was reported as 1.1% and <1% (Moinard-Butot F. et al., 2023), respectively. The summary incidences of grade 3/4 neutropenia induced by sunitinib in patients with gastrointestinal stromal tumors was 9.3% (95% CI, 5.6–13.7%) (Jiang X. et al., 2022). In addition, the RR of sunitinib was reported as 10.39 (95% CI, 1.53–70.72; p = 0.017) compared to the placebo, with heterogeneity not being observed (p < 0.01). Another SR reported that the incidence of CDK 4/6 inhibitor-induced grade 3/4 neutropenia in breast cancer patients ranged from 21.1% to 66% with the calculated RR at 44.00 (95% CI 24.72–78.33; p < 0.0001) (Kassem et al., 2018). Subgroup analyses showed no significant differences in heterogeneity attributed to the dosage of sunitinib and the type of CDK 4/6 inhibitors (palbociclib vs ribociclib vs abemaciclib).

Agranulocytosis and its complications were also identified as an ADE caused by ICIs through SRs of case reports, but they were less commonly reported than all grades of neutropenia (Omar et al., 2020).

Regarding chemotherapy-induced grade 3/4 neutropenia, the median event rate varied significantly with regimen intensity, ranging from single agents at 5.0% to ACVBP-like at 18.0% (Lyman et al., 2016). Notably, the addition of rituximab did not significantly alter the risk characteristics of chemotherapy-related myelosuppression.

#### DIA of the other drugs

In a systematic review of 62 case reports involving 83 patients, antibiotic-induced neutropenia predominantly presented as grade 3/4 severity, accounting for 85% of cases (Holz J.M. et al., 2022). Among these cases, vancomycin and ceftaroline were reported as the most abundant drugs. The lowest ANC caused by non-penicillin β-lactams was significantly lower than that of other classes (p = 0.008, t-statistic = 9.604), with a further significant difference noted between β-lactams and glycopeptides (p = 0.006, t-statistic = 3.099).

In another systematic review of 672 case reports, antibiotic-induced agranulocytosis demonstrated the highest proportion among all the included DIA cases, with 113 cases (16.8%) determined as definite/probable casual relationships (Andersohn F. et al., 2007). This study also showed that antithyroid drugs, psychotropic drugs, and antiarrhythmics were commonly linked to agranulocytosis, with 87, 85, and 39 cases reported for each of them, respectively.

## Discussion

This study presented a disproportionality analysis of DIA utilizing the FAERS database combined with umbrella review. To enhance the reliability of our findings, drug nomenclature was standardized, thereby improving the accuracy and precision of the analysis. Additionally, two disproportionality algorithms were employed to reduce the occurrence of false positives and misidentifications.

An umbrella review was also conducted to corroborate the results of the disproportionality analysis. Specifically, it cross-validated the core results of disproportionality analysis (Carnovale et al., 2021). In our study, these two independent approaches both highlighted anticancer therapies (e.g., PCIs and ICIs) as primary DIA-linked drugs. In addition, several drugs (such as crizotinib and alectinib) were not identified by the disproportionality analysis, and the umbrella review broadened the range of DIA-related drugs beyond the FAERS dataset to reach a fuller profile. The umbrella review also provided richer details, such as cumulative incidence and reduced single-database bias, enhancing the reliability for clinical interpretation (Moore et al., 2024).

Agranulocytosis was obviously a severe ADE, with death and life-threatening outcomes collectively accounting for 18.73% of all reported DIA cases, which closely align with a literature reporting a rate of 16% (Andrès and Maloisel, 2008). In general, anticancer therapy drugs were the primary contributors to agranulocytosis. As shown in Supplementary Table S5, the majority of drugs within the top-eight categories associated with agranulocytosis were drugs used in cancer therapy. Anticancer therapy drugs also occupied the top-five places in both the case number and signal strength and were found to be commonly reported in the umbrella review.

### DIA of anticancer therapy drugs

In clinical practice, myelosuppression was a common adverse effect of chemotherapy drugs (Monnette et al., 2023). In a clinical trial involving venetoclax and the 3 + 7 regimen of daunorubicin and cytarabine, all patients experienced neutropenia or agranulocytosis (Wang et al., 2022). Methotrexate was also noted to cause agranulocytosis even at low doses due to its renal clearance that was susceptible to drug interactions and other various factors (Mayall et al., 1991; Kantarjian et al., 2012).

However, compared to chemotherapy drugs, the hematologic toxicity of targeted therapies, such as monoclonal antibody, ICIs, and PCIs, has been underestimated and still requires more attention (Rattay and Benndorf, 2021; Qin et al., 2024).

Monoclonal antibody (mAb) accounted for the highest proportion in the drug number and case number, which was also reported in one of the included SRs. Rituximab and obinutuzumab, as CD20 inhibitors, contributed to a high proportion of cases. CD20 inhibitors were used to treat B-cell malignancies and have recently been approved for treatment of autoimmune diseases (Lee et al., 2021). Researchers have noted that patients often developed agranulocytosis several months after using CD20 inhibitors, known as late-onset neutropenia (LON) (Shimony et al., 2021). Reported data showed that the median time-to-onset of LON was 175 days (range, 77–204 days) (Pang et al., 2024), which was close to our time-to-onset result at 200.6 days (Supplementary Table S6). LON patients were usually asymptomatic during neutropenia but develop severe fever and infections if agranulocytosis occurs (Shimony et al., 2021; Athni and Barmettler, 2023), especially in pediatric patients (Kamei et al., 2015). This suggested that patients receiving anti-CD20 therapy should have their ANC monitored at specific intervals to prevent and manage LON.

Moreover, toripalimab, an immune checkpoint inhibitor, demonstrated the highest ROR value among all positive signals, thus deserving special attention. A study indicated that agranulocytosis appeared 11 weeks after ICI treatment (Boegeholz et al., 2020), which is earlier than LON caused by CD20 inhibitors.

PCIs were reported in three of the included SRs. Sunitinib and palbociclib were both ranked in the top-50 with respect to case number and were also identified in the umbrella review. No similar phenomenon related to LON was found in PCIs. A study mentioned that grade 3/4 neutropenia was one of the late-onset ADE caused by Nicolini et al. (2015), but the exact onset time was not given for further comparison.

Although the mechanisms remain unclear, a potential explanation for LON induced by CD20 inhibitors includes decreased levels of the neutrophil kinetics regulator SDF-1, disrupted expression of BAFF and APRIL, and apoptosis mediated by effector T-cells (Tesfa et al., 2009; Shimony et al., 2021; Pang et al., 2024). Meanwhile, agranulocytosis caused by ICIs might be antibody-driven or T-cell activation-mediated (Tabchi et al., 2016; Finkel et al., 2020).

### DIA of the other drugs

Unlike that of traditional chemotherapeutic agents, our knowledge of agranulocytosis induced by non-chemotherapy drugs remains limited (Curtis, 2017). Non-chemotherapy drug-induced agranulocytosis is typically idiosyncratic and unpredictable, referred to as idiosyncratic drug-induced agranulocytosis (IDIA) (Andrès et al., 2006). The incidence rate of IDIA is generally regarded as dose-independent, making it difficult to predict the onset using pharmacokinetic principles.

The drugs that were most frequently reported to cause IDIA include antithyroid drugs (ATDs), antipsychotics, antibiotics, anti-epileptics, and antiulcer drugs (Andersohn F. et al., 2007; Garbe, 2007; Johnston and Uetrecht, 2015; Andrès et al., 2017; Curtis, 2017; Wei et al., 2019). These drugs were also identified as positive signals or reported in the umbrella review. Beyond the aforementioned agents, our findings aligned with those of a prior FAERS study on IDIA, which identified antifungals, antigout preparations, antivirals, and antiprotozoals as DIA signals (Wu et al., 2025). Our analysis further expanded this evidence by identifying additional DIA signals, including cyclosporine, tacrolimus, and linezolid. Notably, however, we detected fewer DIA signals among cardiovascular medications such as ticlopidine compared to the previous study. This subtle discrepancy is likely attributed to methodological differences, particularly our use of the SMQ search strategy versus their study focusing on PTs as the search terms.

Deferiprone, one of the identified high-risk drugs, is a second-generation iron chelator that is primarily used to manage chronic iron overload in conditions such as thalassemia and myelodysplastic syndromes (Kontoghiorghes, 2023), with superior oral bioavailability and improved patient adherence compared to the first-generation drugs (Bellotti and Remelli, 2021). However, myelosuppression (especially agranulocytosis) remained a significant safety concern. A clinical study in children with sickle cell disease identified agranulocytosis as a notable adverse effect (Kwiatkowski et al., 2022). Iron was essential for hemoglobin synthesis and cellular proliferation; excessive chelation in the bone marrow disrupts ribonucleotide reductase activity, impairing DNA replication in granulocyte precursors and leading to agranulocytosis (Morales and Xue, 2021). Therefore, a complete blood count was recommended prior to treatment initiation, with weekly monitoring advised during therapy—especially within the high-risk period of 2–4 weeks after onset (Maggio et al., 2020).

Thiamazole is a member of the ATD class and is commonly used in the management of hyperthyroidism, particularly in individuals for whom more aggressive options such as surgery or radioactive iodine therapy are inappropriate (Bartalena, 2013). A recent retrospective cohort study indicated that the incidence of agranulocytosis induced by ATDs was 1.34%. A high incidence rate was observed within the first 72 days of treatment (Kamitani et al., 2023), while our time-to-onset prediction provided a similar result at 65 days. ATD-induced agranulocytosis was followed by a significant reduction in risk for at least the next 6 years, until the ATDs were re-administered (Kamitani et al., 2023).

Both the disproportionality analysis and the umbrella review highlighted the risk of antibiotic-induced agranulocytosis. In a retrospective cohort study, the incidence of antibiotic-induced neutropenia in patients receiving outpatient parenteral antibiotic therapy was 2.2% (95% CI 1.7–2.9) (Lam et al., 2024). Notably, vancomycin, ceftriaxone, and cloxacillin emerged as the most common culprits in this cohort, with the first two also being identified in our study. Similarly, a pediatric-focused systematic review found that ceftriaxone and cloxacillin accounted for a high proportion of antibiotic-induced neutropenia (Battini et al., 2022), whereas vancomycin was less frequently prescribed in children.

The hypotheses for the mechanisms of IDIA were highly diverse, with reactive oxygen species (ROS) hypothesis and hapten hypothesis standing out as the primary ones. In the ROS pathway, activated neutrophils generated ROS, leading to the production of hypochlorous acid (HOCl) (Tesfa et al., 2009). Drugs, such as aminopyrine (the main metabolite of dipyrone) and clozapine, could be oxidized by HOCl into divalent cations, causing neutrophil apoptosis due to ATP and GSH depletion (Uetrecht et al., 1995). In the hapten hypothesis, drugs could be haptens, modifying proteins taken up by antigen-presenting cells and further activating helper T-cells (Parker et al., 1962). Through immune-mediated mechanism, antibodies targeting bone marrow progenitors and cytotoxic T-cells contributed to myelosuppression, ultimately resulting in agranulocytosis. Patients receiving long-term ATD treatment have been detected with increased levels of anti-neutrophil cytoplasmic antibodies (Akamizu et al., 2002; Yoshimura Noh et al., 2024), providing evidence for the hapten hypothesis.

### Limitations

This study has several inherent limitations that warranted acknowledgment. For disproportionality analysis, it was not possible to determine the incidence of DIA events from the FAERS data, and only PS drugs were considered, excluding other suspected drugs. Additionally, our analysis focused solely on individual drugs rather than drug combinations, which turned out to be an inherent limitation in polypharmacy clinical conditions. Given these methodological constraints, we conducted an umbrella review to augment safety evidence synthesis. However, future studies with respect to drug–drug interaction and toxicology of drug combinations are needed to address these aspects and complement the existing findings.

For umbrella review, the dataset included SRs of different research types, which inevitably constrained quantitative synthesis. A major limitation of this umbrella review was the low quality of included SRs, as assessed by AMSTAR-2. SRs failing to rigorously assess RoB could inadvertently include primary studies with high methodological flaws (e.g., unadjusted confounding and small sample sizes), potentially leading to an inaccurate estimation of DIA associations. In addition, inconsistent handling of heterogeneity across SRs limited the comparability of their findings, further complicating the interpretability of our synthesis. SRs should be interpreted with caution, emphasizing the need for future high-quality primary SRs to validate these associations.

While the disproportionality analysis identified associations between drugs and agranulocytosis, it could not establish a causal relationship between them. For instance, positive signals were observed for trilaciclib and colony-stimulating factors (CSFs), which were used to address agranulocytosis. To mitigate this gap as much as possible, we discussed the hypothesis of DIA mechanisms in detail to corroborate the association between drugs and agranulocytosis.

## Conclusion

We integrated disproportionality analysis and umbrella review to provide a spectrum of agranulocytosis-associated drugs. The disproportionality analysis identified 251 drugs with DIA signals, with anticancer therapies being predominant. It revealed 26 high-risk drugs and highlighted age/weight as confounding factors, with the predicted onset time varying. The umbrella review of seven SRs reinforced DIA associations with anticancer therapies and extended to antibiotics, antithyroid drugs, and psychotropics. The DIA drug profile facilitated targeted clinical monitoring and supported evidence-based decision-making. Future research should validate high-risk mechanisms and explore onset-based monitoring protocols to enhance DIA prevention and management.

## Data Availability

The original contributions presented in the study are included in the article/Supplementary Material; further inquiries can be directed to the corresponding author.
