# Supplementary material for: Drug-induced agranulocytosis: a disproportionality analysis and umbrella review
Source: Front Pharmacol. 2025 Sep 17;16:1641747. doi: 10.3389/fphar.2025.1641747 (PMC12484221; doi:10.3389/fphar.2025.1641747)
Supplement: Supplementary file 2 [file Table2.docx]

**Supplementary Table S1. Specific terms for "agranulocytosis" identification**

| **smq_code** | **smq_name_eng** |
| --- | --- |
| 20000023 | Agranulocytosis (SMQ) |
| **pt_code** | **pt_name_eng** |
| 10001507 | Agranulocytosis |
| 10002967 | Aplastic anaemia |
| 10016288 | Febrile neutropenia |
| 10028584 | Myelosuppression |
| 10033661 | Pancytopenia |
| 10049151 | Neutropenic sepsis |
| 10050026 | Panmyelopathy |
| 10053213 | Febrile bone marrow aplasia |
| 10059482 | Neutropenic infection |
| 10062959 | Neutropenic colitis |
| 10065553 | Bone marrow failure |
| 10066274 | Cytopenia |
| 10068043 | Pure white cell aplasia |
| 10071576 | Autoimmune aplastic anaemia |
| 10084828 | Immune-mediated cytopenia |

**Supplementary Table S2. Search Strategy**

| Database | Search Strategy | Results |
| --- | --- | --- |
| PubMed | #1 Agranulocytosis[MeSH])  #2 neutropenia[Title/Abstract]  #3 granulocytopenia[Title/Abstract]  #4 granulopenia[Title/Abstract]  #5 leukopenia[Title/Abstract]  #6 panleukopenia[Title/Abstract]  #7 1 or 2 or 3 or 4 or 5 or 6  #8 systematic review[Filter]  #9 Meta-Analysis[Filter]  #10 8 or 9  #11 (drug-induced[Title/Abstract]) or (induced[Title/Abstract])  #12 7 and 10 and 11 | 183 |
| Embase | #1 *Agranulocytosis/  #2 neutropenia.ab,ti.  #3 granulocytopenia.ab,ti.  #4 granulopenia.ab,ti.  #5 leukopenia.ab,ti.  #6 panleukopenia.ab,ti.  #7 1 or 2 or 3 or 4 or 5 or 6  #8 systematic review.ab,ti.  #9 (meta-analysis or meta analysis or meta-analyses or meta analyses).ab,ti.  #10 8 or 9  #11 (drug-induced or induced).ab,ti.  #12 7 and 10 and 11 | 261 |
| Cochrane Library | #1 MeSH descriptor: [Agranulocytosis] explode all trees  #2 (neutropenia):ti,ab,kw OR (neutropaenia):ti,ab,kw  #3 (granulocytopenia):ti,ab,kw  #4 (granulopenia):ti,ab,kw  #5 (panleukopenia):ti,ab,kw  #6 (leukopenia):ti,ab,kw  #7 #1 OR #2 OR #3 OR #4 OR #5 OR #6  #8 (drug-induced):ti,ab,kw OR (induced):ti,ab,kw  #9 ("systematic review"):ti,ab,kw OR ("meta analysis"):ti,ab,kw OR ("meta analyses"):ti,ab,kw OR ("meta-analysis"):ti,ab,kw OR ("meta-analyses"):ti,ab,kw  #10 #7 AND #8 AND #9 | 21 |
| Web of Science | #1 TS=(agranulocytosis)  #2 TI=(neutropenia OR neutropaenia OR granulocytopenia OR granulopenia OR panleukopenia OR leukopenia) OR AB=(neutropenia OR neutropaenia OR granulocytopenia OR granulopenia OR panleukopenia OR leukopenia)  #3 1 OR 2  #4 TI=(drug-induced OR induced) OR AB=(drug-induced OR induced)  #5 AB=(systematic review OR meta-analysis) OR TI=(systematic review OR meta-analysis)  #6 3 AND 4 AND 5 | 277 |

**Supplementary Table S3. Primary characteristics of agranulocytosis cases in FAERS from 2004 to 2023**

| **Characteristic** | **Case Number (N)** | **Case Proportion (%)** |
| --- | --- | --- |
| **Sex** |  |  |
| Male | 68183 | 42.49 |
| Female | 71422 | 44.50 |
| Unknown | 20882 | 13.01 |
| **Reporter occupation** |  |  |
| Health professional | 137500 | 85.68 |
| Non-health professional | 15482 | 9.65 |
| Unknown | 7505 | 4.68 |
| **Report region** |  |  |
| North America | 50325 | 31.36 |
| South America | 2073 | 1.29 |
| Europe | 58051 | 36.17 |
| Asian | 39143 | 24.39 |
| Oceania | 3078 | 1.92 |
| Africa | 650 | 0.41 |
| Unknown | 7167 | 4.47 |
| **Outcome** |  |  |
| Death | 19679 | 12.26 |
| Life Threatening | 10384 | 6.47 |
| Hospitalization | 41626 | 25.94 |
| Disability | 343 | 0.21 |
| Congenital Anomaly | 13 | 0.01 |
| Required Intervention | 225 | 0.14 |
| Other Serious Events | 32249 | 20.09 |
| Unknown | 55968 | 34.87 |

**Supplementary Table S4. Drugs identified as DIA signals**

| **Drug** | **Category** | **ROR** | **95%CI lower** | **95%CI upper** | **IC** | ***95%CI lower*** | ***95%CI upper*** |
| --- | --- | --- | --- | --- | --- | --- | --- |
| atovaquone | Agents Against Amoebiasis And Other Protozoal Diseases | **7.14** | 5.58 | 9.15 | **2.76** | 1.82 | 3.45 |
| pipobroman | Alkylating Agents | **37.37** | 20.33 | 68.69 | **4.80** | 1.42 | 5.21 |
| ifosfamide | Alkylating Agents | **23.82** | 21.67 | 26.17 | **4.29** | 3.93 | 4.55 |
| thiotepa | Alkylating Agents | **22.07** | 19.12 | 25.48 | **4.20** | 3.63 | 4.57 |
| cyclophosphamide | Alkylating Agents | **18.39** | 17.77 | 19.03 | **3.96** | 3.84 | 4.06 |
| melphalan | Alkylating Agents | **16.75** | 15.56 | 18.04 | **3.86** | 3.60 | 4.08 |
| dacarbazine | Alkylating Agents | **12.88** | 10.20 | 16.27 | **3.54** | 2.59 | 4.12 |
| chlorambucil | Alkylating Agents | **12.33** | 10.09 | 15.07 | **3.48** | 2.69 | 4.01 |
| bendamustine | Alkylating Agents | **11.76** | 10.97 | 12.61 | **3.41** | 3.17 | 3.63 |
| busulfan | Alkylating Agents | **10.78** | 9.86 | 11.79 | **3.30** | 2.98 | 3.57 |
| temozolomide | Alkylating Agents | **9.73** | 9.19 | 10.30 | **3.16** | 2.96 | 3.34 |
| carmustine | Alkylating Agents | **7.44** | 5.74 | 9.64 | **2.81** | 1.82 | 3.53 |
| lomustine | Alkylating Agents | **6.84** | 4.81 | 9.73 | **2.70** | 1.33 | 3.63 |
| mesna | All Other Therapeutic Products | **46.16** | 38.52 | 55.32 | **5.02** | 4.20 | 5.35 |
| dexrazoxane | All Other Therapeutic Products | **42.55** | 33.50 | 54.05 | **4.94** | 3.79 | 5.30 |
| trilaciclib | All Other Therapeutic Products | **16.37** | 10.34 | 25.91 | **3.84** | 1.67 | 4.63 |
| calcium folinate | All Other Therapeutic Products | **12.69** | 11.19 | 14.39 | **3.52** | 3.05 | 3.88 |
| deferiprone | All Other Therapeutic Products | **9.39** | 7.95 | 11.08 | **3.12** | 2.50 | 3.60 |
| calcium levofolinate | All Other Therapeutic Products | **9.05** | 4.75 | 17.26 | **3.07** | 0.28 | 4.37 |
| palifermin | All Other Therapeutic Products | **7.67** | 4.46 | 13.19 | **2.85** | 0.61 | 4.09 |
| amifostine | All Other Therapeutic Products | **4.08** | 2.66 | 6.24 | **1.99** | 0.43 | 3.19 |
| deferasirox | All Other Therapeutic Products | **2.39** | 2.19 | 2.62 | **1.24** | 0.93 | 1.53 |
| tretinoin | Antiacne Preparations For Topical Use | **3.52** | 2.86 | 4.35 | **1.78** | 1.05 | 2.44 |
| dapsone | Antiacne Preparations For Topical Use | **2.77** | 1.97 | 3.90 | **1.45** | 0.26 | 2.49 |
| aprepitant | Antiemetics And Antinauseants | **5.05** | 4.29 | 5.95 | **2.28** | 1.70 | 2.79 |
| palonosetron | Antiemetics And Antinauseants | **3.40** | 2.18 | 5.30 | **1.73** | 0.13 | 3.02 |
| ondansetron | Antiemetics And Antinauseants | **1.50** | 1.29 | 1.75 | **0.58** | 0.07 | 1.08 |
| zonisamide | Antiepileptics | **2.35** | 1.85 | 2.98 | **1.21** | 0.40 | 1.97 |
| carbamazepine | Antiepileptics | **1.36** | 1.20 | 1.53 | **0.43** | 0.02 | 0.84 |
| levetiracetam | Antiepileptics | **1.45** | 1.34 | 1.56 | **0.53** | 0.27 | 0.78 |
| terbinafine | Antifungals For Topical Use | **1.67** | 1.40 | 2.00 | **0.73** | 0.14 | 1.31 |
| colchicine | Antigout Preparations | **5.82** | 4.94 | 6.85 | **2.48** | 1.90 | 2.97 |
| allopurinol | Antigout Preparations | **5.45** | 4.94 | 6.00 | **2.38** | 2.05 | 2.69 |
| colchicine and probenecid | Antigout Preparations | **25.43** | 15.61 | 41.41 | **4.37** | 1.85 | 4.97 |
| chloramphenicol | Antiinfectives | **16.25** | 10.27 | 25.72 | **3.83** | 1.67 | 4.63 |
| trifluridine | Antiinfectives | **3.48** | 3.08 | 3.94 | **1.76** | 1.34 | 2.16 |
| vancomycin | Antiinfectives | **2.22** | 2.01 | 2.45 | **1.13** | 0.80 | 1.46 |
| pyrimethamine | Antimalarials | **5.53** | 3.64 | 8.41 | **2.41** | 0.80 | 3.53 |
| nelarabine | Antimetabolites | **16.17** | 12.48 | 20.95 | **3.82** | 2.72 | 4.41 |
| pemetrexed | Antimetabolites | **14.42** | 13.60 | 15.28 | **3.67** | 3.47 | 3.85 |
| pralatrexate | Antimetabolites | **13.08** | 10.03 | 17.07 | **3.56** | 2.45 | 4.19 |
| fluorouracil | Antimetabolites | **9.65** | 9.15 | 10.17 | **3.15** | 2.96 | 3.32 |
| capecitabine | Antimetabolites | **3.58** | 3.41 | 3.76 | **1.80** | 1.63 | 1.95 |
| cytarabine | Antimetabolites | **43.32** | 41.55 | 45.17 | **4.94** | 4.79 | 5.06 |
| clofarabine | Antimetabolites | **38.63** | 35.23 | 42.36 | **4.84** | 4.48 | 5.07 |
| tioguanine | Antimetabolites | **36.93** | 29.79 | 45.79 | **4.79** | 3.80 | 5.17 |
| decitabine | Antimetabolites | **24.25** | 22.18 | 26.50 | **4.31** | 3.98 | 4.56 |
| azacitidine | Antimetabolites | **20.93** | 20.04 | 21.86 | **4.13** | 3.97 | 4.26 |
| mercaptopurine | Antimetabolites | **19.06** | 17.05 | 21.30 | **4.03** | 3.60 | 4.33 |
| fludarabine | Antimetabolites | **18.75** | 17.59 | 19.98 | **4.00** | 3.77 | 4.19 |
| gemcitabine | Antimetabolites | **8.52** | 8.12 | 8.95 | **2.98** | 2.82 | 3.14 |
| flucytosine | Antimycotics For Systemic Use | **9.63** | 5.34 | 17.39 | **3.16** | 0.58 | 4.35 |
| caspofungin | Antimycotics For Systemic Use | **4.10** | 3.29 | 5.12 | **2.00** | 1.22 | 2.67 |
| posaconazole | Antimycotics For Systemic Use | **3.78** | 3.11 | 4.59 | **1.88** | 1.20 | 2.48 |
| micafungin | Antimycotics For Systemic Use | **3.59** | 2.85 | 4.52 | **1.81** | 1.00 | 2.52 |
| fluconazole | Antimycotics For Systemic Use | **3.04** | 2.68 | 3.44 | **1.57** | 1.15 | 1.98 |
| voriconazole | Antimycotics For Systemic Use | **2.19** | 1.92 | 2.49 | **1.11** | 0.68 | 1.54 |
| albendazole | Antinematodal Agents | **5.94** | 4.44 | 7.94 | **2.51** | 1.42 | 3.33 |
| clozapine | Antipsychotics | **2.52** | 2.41 | 2.63 | **1.30** | 1.15 | 1.45 |
| ticlopidine | Antithrombotic Agents | **9.05** | 7.03 | 11.66 | **3.07** | 2.09 | 3.75 |
| thiamazole | Antithyroid Preparations | **18.12** | 15.81 | 20.77 | **3.97** | 3.43 | 4.33 |
| propylthiouracil | Antithyroid Preparations | **12.19** | 9.22 | 16.11 | **3.46** | 2.31 | 4.14 |
| ursodeoxycholic acid | Bile Therapy | **2.39** | 1.70 | 3.36 | **1.24** | 0.07 | 2.30 |
| dexamethasone | Corticosteroids | **6.77** | 6.49 | 7.07 | **2.67** | 2.52 | 2.81 |
| prednisolone | Corticosteroids | **3.53** | 3.31 | 3.77 | **1.78** | 1.56 | 1.99 |
| prednisone | Corticosteroids | **3.16** | 2.95 | 3.38 | **1.63** | 1.40 | 1.85 |
| hydrocortisone | Corticosteroids | **1.67** | 1.39 | 2.01 | **0.73** | 0.12 | 1.33 |
| methylprednisolone | Corticosteroids | **1.32** | 1.17 | 1.48 | **0.39** | 0.00 | 0.78 |
| idarubicin | Cytotoxic Antibiotics And Related Substances | **38.61** | 33.77 | 44.14 | **4.84** | 4.28 | 5.13 |
| daunorubicin | Cytotoxic Antibiotics And Related Substances | **28.03** | 24.17 | 32.51 | **4.48** | 3.87 | 4.83 |
| mitoxantrone | Cytotoxic Antibiotics And Related Substances | **24.10** | 21.40 | 27.14 | **4.31** | 3.84 | 4.62 |
| dactinomycin | Cytotoxic Antibiotics And Related Substances | **21.05** | 17.47 | 25.37 | **4.15** | 3.37 | 4.59 |
| doxorubicin | Cytotoxic Antibiotics And Related Substances | **18.51** | 17.92 | 19.12 | **3.96** | 3.85 | 4.06 |
| bleomycin | Cytotoxic Antibiotics And Related Substances | **18.14** | 15.66 | 21.00 | **3.97** | 3.39 | 4.35 |
| epirubicin | Cytotoxic Antibiotics And Related Substances | **15.82** | 14.66 | 17.06 | **3.79** | 3.52 | 4.02 |
| ixabepilone | Cytotoxic Antibiotics And Related Substances | **6.97** | 5.59 | 8.69 | **2.72** | 1.90 | 3.36 |
| mitomycin | Cytotoxic Antibiotics And Related Substances | **3.18** | 2.31 | 4.37 | **1.64** | 0.52 | 2.61 |
| ganciclovir | Direct Acting Antivirals | **16.04** | 13.94 | 18.45 | **3.81** | 3.27 | 4.19 |
| valganciclovir | Direct Acting Antivirals | **11.51** | 10.35 | 12.80 | **3.39** | 3.00 | 3.70 |
| foscarnet | Direct Acting Antivirals | **7.30** | 5.46 | 9.75 | **2.79** | 1.67 | 3.57 |
| boceprevir | Direct Acting Antivirals | **3.17** | 2.70 | 3.72 | **1.64** | 1.08 | 2.15 |
| valaciclovir | Direct Acting Antivirals | **3.15** | 2.84 | 3.50 | **1.63** | 1.27 | 1.97 |
| ribavirin | Direct Acting Antivirals | **2.99** | 2.73 | 3.27 | **1.55** | 1.25 | 1.84 |
| zidovudine | Direct Acting Antivirals | **2.78** | 2.35 | 3.29 | **1.45** | 0.88 | 1.99 |
| aciclovir | Direct Acting Antivirals | **2.43** | 2.12 | 2.79 | **1.26** | 0.80 | 1.71 |
| lamivudine | Direct Acting Antivirals | **1.59** | 1.36 | 1.85 | **0.66** | 0.14 | 1.16 |
| pamidronic acid | Drugs Affecting Bone Structure And Mineralization | **3.32** | 2.76 | 3.99 | **1.70** | 1.07 | 2.28 |
| pantoprazole | Drugs For Peptic Ulcer And Gastro-Oesophageal Reflux Disease (Gord) | **1.67** | 1.52 | 1.83 | **0.73** | 0.42 | 1.04 |
| rifabutin | Drugs For Treatment Of Tuberculosis | **3.57** | 2.23 | 5.71 | **1.80** | 0.10 | 3.14 |
| rifampicin | Drugs For Treatment Of Tuberculosis | **2.80** | 2.39 | 3.27 | **1.46** | 0.92 | 1.97 |
| letrozole | Hormone Antagonists And Related Agents | **2.35** | 2.13 | 2.60 | **1.21** | 0.87 | 1.54 |
| fulvestrant | Hormone Antagonists And Related Agents | **2.09** | 1.80 | 2.43 | **1.05** | 0.54 | 1.54 |
| bicalutamide | Hormone Antagonists And Related Agents | **1.92** | 1.49 | 2.48 | **0.93** | 0.07 | 1.75 |
| plerixafor | Immunostimulants | **14.13** | 10.72 | 18.62 | **3.65** | 2.48 | 4.29 |
| filgrastim | Immunostimulants | **11.22** | 10.52 | 11.96 | **3.35** | 3.12 | 3.55 |
| interferon alfa-2a | Immunostimulants | **8.09** | 7.08 | 9.24 | **2.92** | 2.44 | 3.32 |
| aldesleukin | Immunostimulants | **5.50** | 3.71 | 8.15 | **2.40** | 0.90 | 3.47 |
| peginterferon | Immunostimulants | **4.47** | 3.69 | 5.41 | **2.11** | 1.44 | 2.70 |
| pegfilgrastim | Immunostimulants | **3.46** | 3.32 | 3.60 | **1.74** | 1.60 | 1.88 |
| sargramostim | Immunostimulants | **3.16** | 2.11 | 4.75 | **1.63** | 0.19 | 2.84 |
| peginterferon alfa-2b | Immunostimulants | **1.77** | 1.54 | 2.05 | **0.82** | 0.34 | 1.28 |
| peginterferon alfa-2a | Immunostimulants | **1.35** | 1.21 | 1.50 | **0.43** | 0.07 | 0.78 |
| Colony stimulating factors | Immunostimulants | **34.23** | 21.81 | 53.71 | **4.71** | 2.30 | 5.15 |
| globulin,antithymocyte immunoglobulin, lymphocyte immunoglobulin,antithymocyte (equine), rabbit, | Immunosuppressants | **25.78** | 17.06 | 38.95 | **4.39** | 2.30 | 4.95 |
| antithymocyte immunoglobulin (rabbit),antithymocyte immunoglobulin (rabbit) | Immunosuppressants | **15.09** | 8.44 | 26.97 | **3.74** | 0.97 | 4.67 |
| antithymocyte immunoglobulin | Immunosuppressants | **13.65** | 12.50 | 14.91 | **3.61** | 3.29 | 3.87 |
| cabazitaxel | Immunosuppressants | **13.42** | 11.64 | 15.47 | **3.59** | 3.05 | 3.98 |
| vitamin d, pomalidomide | Immunosuppressants | **11.54** | 9.04 | 14.75 | **3.39** | 2.41 | 4.02 |
| vitamin d, lenalidomide | Immunosuppressants | **9.27** | 7.63 | 11.25 | **3.10** | 2.37 | 3.65 |
| azathioprine | Immunosuppressants | **8.64** | 7.82 | 9.56 | **3.01** | 2.65 | 3.32 |
| lymphocyte immunoglobulin, antithymocyte (equine) | Immunosuppressants | **8.18** | 5.25 | 12.76 | **2.94** | 1.11 | 3.99 |
| methotrexate | Immunosuppressants | **6.35** | 6.19 | 6.52 | **2.55** | 2.46 | 2.63 |
| alemtuzumab | Immunosuppressants | **4.16** | 3.77 | 4.59 | **2.01** | 1.67 | 2.33 |
| basiliximab | Immunosuppressants | **4.08** | 3.13 | 5.33 | **1.99** | 1.04 | 2.79 |
| mycophenolic acid | Immunosuppressants | **3.35** | 3.19 | 3.52 | **1.70** | 1.54 | 1.87 |
| cladribine | Immunosuppressants | **3.23** | 2.73 | 3.83 | **1.66** | 1.08 | 2.20 |
| ciclosporin | Immunosuppressants | **2.83** | 2.67 | 3.00 | **1.47** | 1.27 | 1.66 |
| thalidomide | Immunosuppressants | **2.19** | 2.01 | 2.40 | **1.11** | 0.81 | 1.41 |
| eculizumab | Immunosuppressants | **2.17** | 2.03 | 2.31 | **1.10** | 0.88 | 1.31 |
| leflunomide | Immunosuppressants | **1.98** | 1.76 | 2.23 | **0.97** | 0.57 | 1.36 |
| tacrolimus | Immunosuppressants | **1.97** | 1.85 | 2.10 | **0.96** | 0.75 | 1.17 |
| lenalidomide | Immunosuppressants | **1.97** | 1.92 | 2.02 | **0.94** | 0.85 | 1.03 |
| pomalidomide | Immunosuppressants | **1.48** | 1.39 | 1.58 | **0.56** | 0.35 | 0.77 |
| everolimus | Immunosuppressants | **1.36** | 1.24 | 1.50 | **0.44** | 0.13 | 0.75 |
| amphotericin b | Intestinal Antiinfectives | **4.27** | 3.73 | 4.89 | **2.05** | 1.58 | 2.48 |
| sulfasalazine | Intestinal Antiinflammatory Agents | **5.19** | 4.65 | 5.79 | **2.32** | 1.94 | 2.66 |
| mesalazine | Intestinal Antiinflammatory Agents | **1.65** | 1.42 | 1.92 | **0.72** | 0.22 | 1.20 |
| clarithromycin | Macrolides, Lincosamides And Streptogramins | **1.82** | 1.61 | 2.06 | **0.85** | 0.45 | 1.25 |
| toripalimab | Monoclonal Antibodies And Antibody Drug Conjugates | **73.93** | 45.26 | 120.77 | **5.46** | 2.64 | 5.58 |
| gemtuzumab ozogamicin | Monoclonal Antibodies And Antibody Drug Conjugates | **34.25** | 30.95 | 37.90 | **4.71** | 4.31 | 4.96 |
| polatuzumab vedotin | Monoclonal Antibodies And Antibody Drug Conjugates | **16.09** | 13.92 | 18.59 | **3.82** | 3.26 | 4.20 |
| obinutuzumab | Monoclonal Antibodies And Antibody Drug Conjugates | **15.59** | 14.47 | 16.80 | **3.77** | 3.51 | 4.00 |
| pertuzumab | Monoclonal Antibodies And Antibody Drug Conjugates | **14.54** | 13.49 | 15.67 | **3.69** | 3.42 | 3.91 |
| sacituzumab govitecan | Monoclonal Antibodies And Antibody Drug Conjugates | **13.41** | 11.94 | 15.07 | **3.59** | 3.15 | 3.92 |
| brentuximab vedotin | Monoclonal Antibodies And Antibody Drug Conjugates | **13.01** | 12.02 | 14.09 | **3.54** | 3.26 | 3.79 |
| necitumumab | Monoclonal Antibodies And Antibody Drug Conjugates | **12.23** | 8.15 | 18.35 | **3.47** | 1.69 | 4.33 |
| inotuzumab ozogamicin | Monoclonal Antibodies And Antibody Drug Conjugates | **11.56** | 9.30 | 14.36 | **3.40** | 2.53 | 3.97 |
| trastuzumab | Monoclonal Antibodies And Antibody Drug Conjugates | **8.90** | 8.52 | 9.30 | **3.04** | 2.89 | 3.18 |
| enfortumab vedotin | Monoclonal Antibodies And Antibody Drug Conjugates | **8.63** | 7.37 | 10.11 | **3.01** | 2.43 | 3.47 |
| daratumumab | Monoclonal Antibodies And Antibody Drug Conjugates | **8.49** | 7.87 | 9.15 | **2.98** | 2.72 | 3.22 |
| rituximab | Monoclonal Antibodies And Antibody Drug Conjugates | **7.47** | 7.27 | 7.67 | **2.77** | 2.68 | 2.86 |
| tafasitamab | Monoclonal Antibodies And Antibody Drug Conjugates | **6.53** | 4.62 | 9.23 | **2.63** | 1.30 | 3.57 |
| ramucirumab | Monoclonal Antibodies And Antibody Drug Conjugates | **6.28** | 5.44 | 7.26 | **2.58** | 2.07 | 3.02 |
| olaratumab | Monoclonal Antibodies And Antibody Drug Conjugates | **6.06** | 3.81 | 9.62 | **2.53** | 0.72 | 3.72 |
| amivantamab | Monoclonal Antibodies And Antibody Drug Conjugates | **5.98** | 3.77 | 9.50 | **2.52** | 0.71 | 3.70 |
| teclistamab | Monoclonal Antibodies And Antibody Drug Conjugates | **5.91** | 4.50 | 7.75 | **2.50** | 1.49 | 3.28 |
| blinatumomab | Monoclonal Antibodies And Antibody Drug Conjugates | **5.89** | 5.26 | 6.59 | **2.49** | 2.10 | 2.84 |
| atezolizumab | Monoclonal Antibodies And Antibody Drug Conjugates | **5.81** | 5.45 | 6.20 | **2.47** | 2.25 | 2.68 |
| isatuximab | Monoclonal Antibodies And Antibody Drug Conjugates | **5.56** | 4.47 | 6.92 | **2.41** | 1.62 | 3.06 |
| elotuzumab | Monoclonal Antibodies And Antibody Drug Conjugates | **5.39** | 4.32 | 6.73 | **2.37** | 1.57 | 3.03 |
| bevacizumab | Monoclonal Antibodies And Antibody Drug Conjugates | **5.19** | 5.00 | 5.39 | **2.30** | 2.17 | 2.42 |
| dinutuximab beta | Monoclonal Antibodies And Antibody Drug Conjugates | **5.03** | 2.94 | 8.60 | **2.28** | 0.22 | 3.67 |
| trastuzumab deruxtecan | Monoclonal Antibodies And Antibody Drug Conjugates | **5.03** | 4.38 | 5.78 | **2.28** | 1.79 | 2.71 |
| docetaxel | Monoclonal Antibodies And Antibody Drug Conjugates | **4.86** | 4.66 | 5.08 | **2.22** | 2.07 | 2.36 |
| durvalumab | Monoclonal Antibodies And Antibody Drug Conjugates | **4.83** | 4.36 | 5.35 | **2.22** | 1.86 | 2.55 |
| panitumumab | Monoclonal Antibodies And Antibody Drug Conjugates | **4.66** | 4.20 | 5.16 | **2.17** | 1.82 | 2.49 |
| cetuximab | Monoclonal Antibodies And Antibody Drug Conjugates | **4.56** | 4.25 | 4.89 | **2.14** | 1.90 | 2.36 |
| mosunetuzumab | Monoclonal Antibodies And Antibody Drug Conjugates | **4.29** | 2.56 | 7.20 | **2.06** | 0.13 | 3.46 |
| mogamulizumab | Monoclonal Antibodies And Antibody Drug Conjugates | **4.17** | 3.10 | 5.62 | **2.02** | 0.95 | 2.90 |
| pembrolizumab | Monoclonal Antibodies And Antibody Drug Conjugates | **3.45** | 3.26 | 3.65 | **1.74** | 1.55 | 1.93 |
| trastuzumab emtansine | Monoclonal Antibodies And Antibody Drug Conjugates | **3.24** | 2.72 | 3.87 | **1.67** | 1.06 | 2.23 |
| ofatumumab | Monoclonal Antibodies And Antibody Drug Conjugates | **1.58** | 1.41 | 1.76 | **0.65** | 0.28 | 1.01 |
| nivolumab | Monoclonal Antibodies And Antibody Drug Conjugates | **1.55** | 1.45 | 1.65 | **0.62** | 0.39 | 0.84 |
| ipilimumab | Monoclonal Antibodies And Antibody Drug Conjugates | **1.44** | 1.26 | 1.65 | **0.52** | 0.07 | 0.96 |
| lestaurtinib | Na | **37.69** | 23.67 | 60.03 | **4.81** | 2.28 | 5.21 |
| azithromycin,methotrexate | Na | **14.83** | 7.65 | 28.74 | **3.72** | 0.55 | 4.72 |
| calaspargase pegol | Na | **5.81** | 3.17 | 10.67 | **2.48** | 0.08 | 3.94 |
| metamizole sodium | Other Analgesics And Antipyretics | **36.71** | 22.61 | 59.59 | **4.79** | 2.14 | 5.20 |
| linezolid | Other Antibacterials | **9.02** | 8.44 | 9.64 | **3.06** | 2.83 | 3.27 |
| daptomycin | Other Antibacterials | **2.28** | 1.96 | 2.66 | **1.17** | 0.66 | 1.67 |
| cytarabine and daunorubicin | Other Antineoplastic Agents | **34.72** | 30.20 | 39.92 | **4.72** | 4.14 | 5.04 |
| eribulin | Other Antineoplastic Agents | **26.32** | 24.22 | 28.59 | **4.41** | 4.10 | 4.64 |
| vorinostat | Other Antineoplastic Agents | **19.23** | 16.61 | 22.27 | **4.04** | 3.46 | 4.42 |
| pegaspargase | Other Antineoplastic Agents | **18.97** | 17.36 | 20.73 | **4.02** | 3.69 | 4.27 |
| cisplatin | Other Antineoplastic Agents | **15.53** | 14.85 | 16.25 | **3.76** | 3.60 | 3.90 |
| carboplatin | Other Antineoplastic Agents | **14.14** | 13.70 | 14.60 | **3.62** | 3.51 | 3.72 |
| tisagenlecleucel | Other Antineoplastic Agents | **12.55** | 11.07 | 14.24 | **3.50** | 3.03 | 3.86 |
| talazoparib | Other Antineoplastic Agents | **12.33** | 9.86 | 15.42 | **3.48** | 2.58 | 4.05 |
| glasdegib | Other Antineoplastic Agents | **11.82** | 8.11 | 17.24 | **3.43** | 1.80 | 4.26 |
| idecabtagene vicleucel | Other Antineoplastic Agents | **11.33** | 8.60 | 14.92 | **3.37** | 2.24 | 4.05 |
| arsenic trioxide | Other Antineoplastic Agents | **11.31** | 9.29 | 13.77 | **3.37** | 2.60 | 3.90 |
| axicabtagene ciloleucel | Other Antineoplastic Agents | **9.58** | 8.68 | 10.58 | **3.15** | 2.79 | 3.45 |
| panobinostat | Other Antineoplastic Agents | **8.94** | 7.26 | 11.01 | **3.06** | 2.26 | 3.64 |
| venetoclax | Other Antineoplastic Agents | **8.80** | 8.45 | 9.16 | **3.02** | 2.88 | 3.15 |
| pentostatin | Other Antineoplastic Agents | **8.69** | 5.91 | 12.78 | **3.02** | 1.45 | 3.96 |
| lurbinectedin | Other Antineoplastic Agents | **8.35** | 6.12 | 11.39 | **2.97** | 1.74 | 3.77 |
| omacetaxine mepesuccinate | Other Antineoplastic Agents | **7.69** | 5.64 | 10.48 | **2.86** | 1.65 | 3.68 |
| brexucabtagene autoleucel | Other Antineoplastic Agents | **7.69** | 5.89 | 10.03 | **2.85** | 1.83 | 3.58 |
| romidepsin | Other Antineoplastic Agents | **7.52** | 5.91 | 9.56 | **2.82** | 1.91 | 3.50 |
| asparaginase | Other Antineoplastic Agents | **7.39** | 6.35 | 8.61 | **2.80** | 2.25 | 3.26 |
| denileukin diftitox | Other Antineoplastic Agents | **7.16** | 3.89 | 13.19 | **2.76** | 0.25 | 4.13 |
| olaparib | Other Antineoplastic Agents | **5.24** | 4.81 | 5.72 | **2.33** | 2.03 | 2.61 |
| hydroxycarbamide | Other Antineoplastic Agents | **5.07** | 4.34 | 5.93 | **2.29** | 1.74 | 2.77 |
| oxaliplatin | Other Antineoplastic Agents | **4.98** | 4.71 | 5.27 | **2.26** | 2.06 | 2.44 |
| carfilzomib | Other Antineoplastic Agents | **4.63** | 4.24 | 5.06 | **2.16** | 1.86 | 2.44 |
| bortezomib | Other Antineoplastic Agents | **4.63** | 4.37 | 4.90 | **2.15** | 1.96 | 2.34 |
| selinexor | Other Antineoplastic Agents | **3.33** | 2.84 | 3.91 | **1.70** | 1.16 | 2.21 |
| ivosidenib | Other Antineoplastic Agents | **3.27** | 2.37 | 4.52 | **1.68** | 0.54 | 2.66 |
| mitotane | Other Antineoplastic Agents | **3.21** | 2.06 | 5.00 | **1.65** | 0.07 | 2.95 |
| enasidenib | Other Antineoplastic Agents | **3.12** | 2.51 | 3.87 | **1.61** | 0.87 | 2.29 |
| ixazomib | Other Antineoplastic Agents | **2.73** | 2.47 | 3.03 | **1.42** | 1.08 | 1.76 |
| niraparib | Other Antineoplastic Agents | **2.23** | 2.01 | 2.47 | **1.14** | 0.79 | 1.47 |
| rucaparib | Other Antineoplastic Agents | **2.04** | 1.74 | 2.40 | **1.02** | 0.47 | 1.54 |
| ceftaroline fosamil | Other Beta-Lactam Antibacterials | **8.22** | 5.96 | 11.33 | **2.94** | 1.67 | 3.78 |
| meropenem | Other Beta-Lactam Antibacterials | **6.76** | 5.94 | 7.69 | **2.68** | 2.22 | 3.07 |
| bacteriostatic sodium chloride, sodium chloride, | Other Beta-Lactam Antibacterials | **6.75** | 3.67 | 12.42 | **2.68** | 0.20 | 4.08 |
| piperacillin, tazobactam | Other Beta-Lactam Antibacterials | **5.46** | 4.92 | 6.05 | **2.39** | 2.03 | 2.71 |
| cefotaxime | Other Beta-Lactam Antibacterials | **5.18** | 3.88 | 6.92 | **2.32** | 1.25 | 3.16 |
| doripenem | Other Beta-Lactam Antibacterials | **4.60** | 2.94 | 7.20 | **2.15** | 0.48 | 3.38 |
| ceftazidime | Other Beta-Lactam Antibacterials | **4.09** | 2.82 | 5.92 | **1.99** | 0.64 | 3.06 |
| imipenem and cilastatin | Other Beta-Lactam Antibacterials | **2.31** | 1.82 | 2.94 | **1.19** | 0.38 | 1.95 |
| ceftriaxone | Other Beta-Lactam Antibacterials | **2.18** | 1.89 | 2.52 | **1.11** | 0.63 | 1.57 |
| vincristine | Plant Alkaloids And Other Natural Products | **31.48** | 29.77 | 33.30 | **4.61** | 4.40 | 4.77 |
| trabectedin | Plant Alkaloids And Other Natural Products | **23.62** | 20.41 | 27.33 | **4.29** | 3.69 | 4.64 |
| etoposide | Plant Alkaloids And Other Natural Products | **23.10** | 21.97 | 24.28 | **4.25** | 4.07 | 4.40 |
| algestone,etoposide | Plant Alkaloids And Other Natural Products | **20.53** | 10.44 | 40.40 | **4.12** | 0.67 | 4.92 |
| topotecan | Plant Alkaloids And Other Natural Products | **17.96** | 16.30 | 19.78 | **3.95** | 3.59 | 4.23 |
| vinorelbine | Plant Alkaloids And Other Natural Products | **15.64** | 13.83 | 17.69 | **3.78** | 3.31 | 4.12 |
| vinblastine | Plant Alkaloids And Other Natural Products | **14.02** | 8.89 | 22.09 | **3.64** | 1.56 | 4.50 |
| irinotecan | Plant Alkaloids And Other Natural Products | **7.48** | 6.96 | 8.04 | **2.81** | 2.56 | 3.04 |
| paclitaxel | Plant Alkaloids And Other Natural Products | **6.17** | 5.88 | 6.48 | **2.55** | 2.38 | 2.70 |
| gilteritinib | Protein Kinase Inhibitors | **38.06** | 34.63 | 41.82 | **4.82** | 4.45 | 5.06 |
| midostaurin | Protein Kinase Inhibitors | **12.16** | 10.59 | 13.95 | **3.46** | 2.94 | 3.85 |
| asciminib | Protein Kinase Inhibitors | **11.87** | 7.29 | 19.31 | **3.43** | 1.26 | 4.40 |
| zanubrutinib | Protein Kinase Inhibitors | **7.71** | 6.03 | 9.86 | **2.86** | 1.92 | 3.54 |
| copanlisib | Protein Kinase Inhibitors | **7.19** | 4.10 | 12.61 | **2.77** | 0.46 | 4.06 |
| ponatinib | Protein Kinase Inhibitors | **4.81** | 4.33 | 5.35 | **2.21** | 1.85 | 2.55 |
| temsirolimus | Protein Kinase Inhibitors | **4.05** | 3.35 | 4.88 | **1.98** | 1.32 | 2.56 |
| pralsetinib | Protein Kinase Inhibitors | **3.58** | 2.49 | 5.16 | **1.81** | 0.50 | 2.88 |
| idelalisib | Protein Kinase Inhibitors | **3.51** | 3.03 | 4.07 | **1.78** | 1.27 | 2.25 |
| palbociclib | Protein Kinase Inhibitors | **3.36** | 3.21 | 3.50 | **1.70** | 1.56 | 1.84 |
| abemaciclib | Protein Kinase Inhibitors | **2.98** | 2.63 | 3.37 | **1.55** | 1.12 | 1.95 |
| imatinib | Protein Kinase Inhibitors | **2.69** | 2.54 | 2.84 | **1.40** | 1.21 | 1.58 |
| osimertinib | Protein Kinase Inhibitors | **2.47** | 2.24 | 2.72 | **1.28** | 0.95 | 1.60 |
| sunitinib | Protein Kinase Inhibitors | **2.27** | 2.11 | 2.44 | **1.16** | 0.92 | 1.40 |
| nilotinib | Protein Kinase Inhibitors | **1.98** | 1.81 | 2.17 | **0.97** | 0.66 | 1.28 |
| sorafenib | Protein Kinase Inhibitors | **1.86** | 1.66 | 2.08 | **0.88** | 0.51 | 1.25 |
| lapatinib | Protein Kinase Inhibitors | **1.82** | 1.58 | 2.09 | **0.85** | 0.38 | 1.31 |
| ribociclib | Protein Kinase Inhibitors | **1.80** | 1.58 | 2.04 | **0.83** | 0.41 | 1.25 |
| ruxolitinib | Protein Kinase Inhibitors | **1.71** | 1.60 | 1.83 | **0.76** | 0.54 | 0.98 |
| dasatinib | Protein Kinase Inhibitors | **1.41** | 1.27 | 1.57 | **0.49** | 0.14 | 0.84 |
| sulfamethoxazole and trimethoprim | Sulfonamides And Trimethoprim | **4.27** | 3.87 | 4.72 | **2.05** | 1.71 | 2.37 |
| trimethoprim | Sulfonamides And Trimethoprim | **2.98** | 2.23 | 4.00 | **1.55** | 0.53 | 2.45 |
| ibritumomab tiuxetan (^90^Y) | Therapeutic Radiopharmaceuticals | **22.77** | 19.20 | 27.01 | **4.24** | 3.53 | 4.65 |
| strontium (^89^Sr) chloride | Therapeutic Radiopharmaceuticals | **17.63** | 12.26 | 25.34 | **3.93** | 2.26 | 4.61 |
| samarium sm-153 | Therapeutic Radiopharmaceuticals | **15.08** | 8.24 | 27.59 | **3.74** | 0.85 | 4.69 |
| tositumomab/iodine (^131^I) tositumomab | Therapeutic Radiopharmaceuticals | **8.57** | 4.64 | 15.85 | **3.00** | 0.38 | 4.29 |
| radium (^223^Ra) dichloride | Therapeutic Radiopharmaceuticals | **5.76** | 5.02 | 6.62 | **2.46** | 1.97 | 2.89 |
| folic acid | Vitamin B12 And Folic Acid | **5.90** | 4.94 | 7.05 | **2.50** | 1.86 | 3.03 |
| eltrombopag | Vitamin K And Other Hemostatics | **2.42** | 2.21 | 2.66 | **1.25** | 0.94 | 1.56 |
| romiplostim | Vitamin K And Other Hemostatics | **1.90** | 1.65 | 2.18 | **0.91** | 0.45 | 1.36 |

Na: ATC code not applicable

**Supplementary Table S5. Classification of positive drugs** **according to the WHO ATC system**

| **WHO ATC Category** | **DIA cases numbers** | **Number of Drugs** | **Drugs** |
| --- | --- | --- | --- |
| Monoclonal antibodies and antibody drug conjugates | 21524 | 35 | Toripalimab, Gemtuzumab Ozogamicin,  Polatuzumab Vedotin, Obinutuzumab,  Pertuzumab, Sacituzumab Govitecan,  Brentuximab Vedotin, Necitumumab, Inotuzumab Ozogamicin, Trastuzumab, Enfortumab Vedotin, Daratumumab, Rituximab, Tafasitamab, Ramucirumab, Olaratumab, Amivantamab, Teclistamab, Blinatumomab, Atezolizumab, Isatuximab, Elotuzumab, Bevacizumab, Dinutuximab beta, Trastuzumab Deruxtecan, Durvalumab, Panitumumab, Cetuximab, Mosunetuzumab, Mogamulizumab, Pembrolizumab, Trastuzumab Emtansine, Ofatumumab, Nivolumab, Ipilimumab |
| Immunosuppressants | 21099 | 21 | Antithymocyte Immunoglobulin, Pomalidomide and Vitamin D, Azathioprine, Methotrexate, Alemtuzumab, Basiliximab, Mycophenolic acid, Cladribine, Ciclosporin, Thalidomide, Eculizumab, Leflunomide, Lenalidomide, Cabazitaxel, Pomalidomide, Everolimus, Tacrolimus, Lenalidomide and Vitamin D, [Antithymocyte immunoglobulin (rabbit), antithymocyte immunoglobulin (rabbit)], [Globulin, antithymocyte immunoglobulin, lymphocyte immunoglobulin, antithymocyte (equine), rabbit], [Lymphocyte immunoglobulin, antithymocyte (equine)] |
| Other antineoplastic agents | 17345 | 33 | Cytarabine and Daunorubicin, Eribulin, Vorinostat, Pegaspargase, Cisplatin, Carboplatin, Tisagenlecleucel, Talazoparib, Glasdegib, Idecabtagene Vicleucel, Arsenic Trioxide, Axicabtagene Ciloleucel, Panobinostat, Venetoclax, Pentostatin, Lurbinectedin, Omacetaxine Mepesuccinate, Brexucabtagene Autoleucel, Romidepsin, Asparaginase, Denileukin Diftitox, Olaparib, Hydroxycarbamide, Oxaliplatin, Carfilzomib, Bortezomib, Selinexor, Ivosidenib, Mitotane, Enasidenib, Ixazomib, Niraparib, Rucaparib |
| Antimetabolites | 14869 | 13 | Cytarabine, Clofarabine, Tioguanine, Decitabine, Azacitidine, Mercaptopurine, Fludarabine, Nelarabine, Pemetrexed, Pralatrexate, Fluorouracil, Gemcitabine, Capecitabine |
| Plant alkaloids and other natural products | 9230 | 11 | Vincristine, Trabectedin, Etoposide, Topotecan, Vinorelbine, Vinblastine, Irinotecan, Paclitaxel, Docetaxel, Algestone And Etoposide |
| Protein kinase inhibitors | 8912 | 20 | Gilteritinib, Midostaurin, Asciminib, Zanubrutinib, Copanlisib, Ponatinib, Temsirolimus, Pralsetinib, Idelalisib, Palbociclib, Abemaciclib, Imatinib, Osimertinib, Sunitinib, Nilotinib, Sorafenib, Lapatinib, Ribociclib, Ruxolitinib, Dasatinib |
| Alkylating agents | 8554 | 12 | Pipobroman, Ifosfamide, Thiotepa, Cyclophosphamide, Melphalan, Dacarbazine, Chlorambucil, Bendamustine, Busulfan, Temozolomide, Carmustine, Lomustine |
| Cytotoxic antibiotics and related substances | 6472 | 9 | Idarubicin, Daunorubicin, Mitoxantrone, Dactinomycin, Doxorubicin, Bleomycin, Epirubicin, Ixabepilone, Mitomycin, |
| Corticosteroids | 4434 | 5 | Dexamethasone, Prednisolone, Hydrocortisone, Prednisone, Methylprednisolone |
| Immunostimulants | 4409 | 10 | Colony stimulating factors, Plerixafor, Filgrastim, Interferon alfa-2a, Aldesleukin, Peginterferon, Pegfilgrastim, Sargramostim, Peginterferon alfa-2b, Peginterferon alfa-2a |
| Direct acting antivirals | 2161 | 9 | Ganciclovir, Valganciclovir, Foscarnet, Boceprevir, Valaciclovir, Ribavirin, Zidovudine, Acyclovir, Lamivudine |
| Antipsychotics | 2069 | 1 | Clozapine |
| All other therapeutic products | 1235 | 9 | Deferiprone, Mesna, Dexrazoxane, Trilaciclib, Calcium Folinate, Calcium Levofolinate, Deferasirox, Palifermin, Amifostine |
| Other antibacterials | 1113 | 2 | Linezolid, Daptomycin |
| Other beta-lactam antibacterials | 1041 | 9 | Ceftaroline Fosamil, Meropenem, Cefotaxime, Doripenem, Ceftazidime, Imipenem and Cilastatin, Ceftriaxone, Bacteriostatic Sodium Chloride, Piperacillin and Tazobactam |
| Antiepileptics | 966 | 3 | Levetiracetam, Carbamazepine, Zonisamide |
| Antimycotics for systemic use | 758 | 6 | Flucytosine, Caspofungin, Posaconazole, Micafungin, Fluconazole, Voriconazole |
| Antiinfectives | 678 | 3 | Chloramphenicol, Trifluridine, Vancomycin |
| Vitamin k and other hemostatics | 660 | 2 | Eltrombopag, Romiplostim |
| Hormone antagonists and related agents | 623 | 3 | Letrozole, Fulvestrant, Bicalutamide |
| Antigout preparations | 598 | 3 | Colchicine and Probenecid, Colchicine, Allopurinol |
| Intestinal antiinflammatory agents | 511 | 2 | Sulfasalazine, Mesalazine |
| Sulfonamides and trimethoprim | 459 | 2 | Sulfamethoxazole and Trimethoprim, Trimethoprim |
| Drugs for peptic ulcer and gastro-oesophageal reflux disease (gord) | 454 | 1 | Pantoprazole |
| Therapeutic radiopharmaceuticals | 429 | 5 | Samarium (^153^Sm), Strontium (^89^Sr) Chloride, Radium (^223^Ra) Dichloride, Ibritumomab Tiuxetan (^90^Y), Tositumomab/Iodine (^131^I), |
| Antiemetics and antinauseants | 335 | 3 | Aprepitant, Palonosetron, Ondansetron |
| Antithyroid preparations | 296 | 2 | Thiamazole, Propylthiouracil |
| Macrolides, lincosamides and streptogramins | 266 | 1 | Clarithromycin |
| Intestinal antiinfectives | 217 | 1 | Amphotericin B |
| Drugs for treatment of tuberculosis | 176 | 2 | Rifabutin, Rifampicin |
| Vitamin b12 and folic acid | 128 | 1 | Folic acid |
| Antiacne preparations for topical use | 124 | 2 | Tretinoin, Dapsone |
| Antifungals for topical use | 124 | 1 | Terbinafine |
| Drugs affecting bone structure and mineralization | 118 | 1 | Pamidronic acid |
| Agents against amoebiasis and other protozoal diseases | 67 | 1 | Atovaquone |
| Antithrombotic agents | 65 | 1 | Ticlopidine |
| Antinematodal agents | 48 | 1 | Albendazole |
| NA | 45 | 3 | Lestaurtinib*  Calaspargase Pegol*  Azithromycin,Methotrexate* |
| Bile therapy | 34 | 1 | Ursodeoxycholic acid |
| Antimalarials | 23 | 1 | Pyrimethamine |
| Other analgesics and antipyretics | 22 | 1 | Metamizole Sodium |

**Supplementary Table S6. Time-to-onset time of DIA in different drugs**

| **Drug** | **Mean** | **Q1** | **Median** | **Q3** |
| --- | --- | --- | --- | --- |
| Amlodipine | 91.5 | 10 | 12 | 60 |
| Amoxicillin | 10.9 | 3 | 8 | 15.5 |
| Azithromycin | 8.1 | 2 | 4 | 6 |
| Carboplatin | 18 | 6 | 8 | 19 |
| Clozapine | 1121.3 | 51 | 144.5 | 1331 |
| Cyclophosphamide | 32.3 | 7 | 8 | 11 |
| Daptomycin | 22.1 | 11 | 18 | 33 |
| Deferiprone | 407.4 | 40.3 | 108.5 | 235.5 |
| Docetaxel | 15.1 | 6 | 7 | 9 |
| Doxorubicin | 32.6 | 8.3 | 13 | 42 |
| Ibuprofen | 28.7 | 2 | 6 | 13 |
| Lamotrigine | 73.7 | 13 | 29 | 45 |
| Lansoprazole | 33.6 | 12 | 30 | 41 |
| Levetiracetam | 55.3 | 14.5 | 23 | 41 |
| Methotrexate | 335.8 | 6.5 | 12 | 141.3 |
| Mycophenolic acid | 289.8 | 41 | 82 | 160.5 |
| Olanzapine | 124.8 | 10 | 19 | 28.5 |
| Pantoprazole | 91.8 | 12 | 23 | 32 |
| Quetiapine | 116.1 | 5 | 20 | 58.5 |
| Rituximab | 200.6 | 22 | 75 | 227.5 |
| Sodium chloride | 9 | 7 | 8 | 10 |
| Sulfasalazine | 48.2 | 28 | 44 | 53.8 |
| Thiamazole | 65 | 30.3 | 41.5 | 65.8 |
| Valaciclovir | 52.6 | 4 | 20 | 79 |
| Vancomycin | 15.9 | 10 | 16 | 20.3 |
| Venetoclax | 35.9 | 6 | 10.5 | 21.5 |

**Supplementary Table S7. List of excluded records with specific reasons**

| Search Strategy | Reason |
| --- | --- |
| 1 Eiger D., Agostinetto E., Caparica R., *et al.* 44P The hematologic toxicities of chemo-immunotherapy compared to chemotherapy alone: A systematic review and meta-analysis. *Ann Oncol* 2020; 31: S1433 EP-S1434.  2 Bashir MO, Wu S. E 19729 Docetaxel-induced neutropenia and neutropenic fever in general and elderly patient population: A meta-analysis. *JOURNAL OF CLINICAL ONCOLOGY* 2011; 29. DOI:10.1200/jco.2011.29.15_suppl.e19729.  3 Andersohn F, Konzen C, Garbe E. Non-chemotherapy drug-induced agranulocytosis: A systematic review of case reports. *PHARMACOEPIDEMIOLOGY AND DRUG SAFETY* 2007; 16: S52–S52.  4 Gisbert JP, Gomollon F. S1240 Azathioprine (AZA) and mercaptopurine (MP)-induced myelotoxicity in patients with inflammatory bowel disease (IBD): A systematic review. *GASTROENTEROLOGY* 2008; 134: A208–A208.  5 Konte B., Walters J.T., Giegling I., *et al.* SU102 META-ANALYSIS OF CLOZAPINE-ASSOCIATED NEUTROPENIA AND AGRANULOCYTOSIS. *Eur Neuropsychopharmacol* 2019; 29: S1320 EP-S1321.  6 Andersohn F, Konzen C, Garbe E. Systematic review of case reports: Nonchemotherapy drug-induced agranulocytosis in children. *PHARMACOEPIDEMIOLOGY AND DRUG SAFETY* 2008; 17: S152–3.  7 Andersohn F, Konzen C, Garbe E. Systematic review: non-chemotherapy drug-induced agranulocytosis in children. *BASIC & CLINICAL PHARMACOLOGY & TOXICOLOGY* 2007; 101: 389–389.  8 Munshi T., Naeem F., Ayub M., *et al.* The haematological side effects of clozapine: literature review and meta-analysis. *BJPsych Int* 2021; 7: S274. | Meeting abstract or Poster |
| 9 Battini V., Mari A., Gringeri M., *et al.* Antibiotic-Induced Neutropenia in Pediatric Patients: New Insights From Pharmacoepidemiological Analyses and a Systematic Review. *Front Pharmacol* 2022; 13: 877932.  10 Sim B.L., Sim B.Z., Tunbridge M., Liew D.F.L., Robinson P.C. Examining the Characteristics of Colchicine-Induced Myelosuppression in Clinical Cases: A Systematic Review. *J Rheumatol* 2023; 50: 400 EP – 407.  11 Wang Q., He Z., Wu X., Wei Y., Huang J. Hematologic adverse effects induced by piperacillin-tazobactam: a systematic review of case reports. *Int J Clin Pharm* 2020; 42: 1026 EP – 1035.  12 Scheetz M.H., McKoy J.M., Parada J.P., *et al.* Systematic review of piperacillin-induced neutropenia. *Drug Saf* 2007; 30: 295 EP – 306. | Grade 1/2 neutropenia |

**Supplementary Table S8. AMSTAR-2 assessments for included systematic reviews**

|  | **Julian, 2022** | **Fabien, 2023** | **Nabil, 2020** | **Jiang, 2022** | **Loay, 2018** | **Gary H, 2016** | **Andersohn, 2007** |
| --- | --- | --- | --- | --- | --- | --- | --- |
| **Item1** | Yes | Yes | Yes | Yes | Yes | Yes | Yes |
| **Item2** | No | No | No | Yes | Partial Yes | No | Partial Yes |
| **Item3** | No | No | Yes | Yes | Yes | Yes | Yes |
| **Item4** | No | No | Partial Yes | Partial Yes | Partial Yes | No | Partial Yes |
| **Item5** | Yes | No | Yes | Yes | Yes | Yes | Yes |
| **Item6** | Yes | No | Yes | Yes | Yes | Yes | Yes |
| **Item7** | Partial Yes | Partial Yes | Partial Yes | Partial Yes | Partial Yes | Partial Yes | Partial Yes |
| **Item8** | Yes | Yes | Yes | Yes | Yes | Yes | Yes |
| **Item9** | No | No | No | Partial Yes | No | No | No |
| **Item10** | No | No | No | No | No | No | No |
| **Item11** | NA | NA | NA | Yes | Yes | NA | NA |
| **Item12** | NA | NA | NA | Partial Yes | No | NA | NA |
| **Item13** | No | No | No | Yes | No | Yes | Yes |
| **Item14** | Partial Yes | Partial Yes | Partial Yes | Yes | Yes | Yes | Partial Yes |
| **Item15** | No | No | No | Yes | No | No | No |
| **Item16** | Yes | Yes | Yes | Yes | Yes | Yes | Yes |
| **Quality** | Critically Low | Critically Low | Low | Moderate | Low | Low | Low |
